# Priority setting in health: development and application of a multi-criteria algorithm for the population of New Zealand’s Waikato region

**DOI:** 10.1186/s12962-018-0121-z

**Published:** 2018-11-09

**Authors:** Rashmi Dayalu, Elizabeth T. Cafiero-Fonseca, Victoria Y. Fan, Heather Schofield, David E. Bloom

**Affiliations:** 1 Department of Global Health and Population, Harvard T.H. Chan School of Public Health, Boston, USA; 2 Performance Analysis and Improvement, Massachusetts General Hospital/Massachusetts General Physicians Organization, Boston, USA; 3 Office of Public Health Studies, Myron B. Thompson School of Social Work, University of Hawai‘i at Mānoa, Honolulu, USA; 4 François-Xavier Bagnoud Center for Health and Human Rights, Harvard T.H. Chan School of Public Health, Boston, USA; 5 Department of Medical Ethics and Health Policy, The Perelman School of Medicine, The University of Pennsylvania, Philadelphia, USA; 6 The Wharton School, The University of Pennsylvania, Philadelphia, USA

**Keywords:** Multi-criteria, Health, Priority setting, Income, Equity, Cost-effectiveness, Multimorbidity, Aging, Public, Preference elicitation

## Abstract

**Background:**

Priority setting in a climate of diverse needs and limited resources is one of the most significant challenges faced by health care policymakers. This paper develops and applies a comprehensive multi-criteria algorithm to help determine the relative importance of health conditions that affect a defined population.

**Methods:**

Our algorithm is implemented in the context of the Waikato District Health Board (WDHB) in New Zealand, which serves approximately 10% of the New Zealand population. Strategic priorities of the WDHB are operationalized into five criteria along which the algorithm is structured—scale of disease, household financial impact of disease, health equity, cost-effectiveness, and multimorbidity burden. Using national-level data and published literature from New Zealand, the World Health Organization, and other high-income Commonwealth countries, 25 health conditions in Waikato are identified and mapped to these five criteria. These disease-criteria mappings are weighted with data from an ordered choice survey administered to the general public of the Waikato region. The resulting output of health conditions ranked in order of relative importance is validated against an explicit list of health concerns, provided by the survey respondents.

**Results:**

Heart disease and cancerous disorders are assigned highest priority rankings according to both the algorithm and the survey data, suggesting that our model is aligned with the primary health concerns of the general public. All five criteria are weighted near-equal across survey respondents, though the average health equity preference score is 9.2% higher for Māori compared to non-Māori respondents. Older respondents (50 years and above) ranked issues of multimorbidity 4.2% higher than younger respondents.

**Conclusions:**

Health preferences of the general population can be elicited using ordered-choice surveys and can be used to weight data for health conditions across multiple criteria, providing policymakers with a practical tool to inform which health conditions deserve the most attention. Our model connects public health strategic priorities, the health impacts and financial costs of particular health conditions, and the underlying preferences of the general public. We illustrate a practical approach to quantifying the foundational criteria that drive public preferences, for the purpose of relevant, legitimate, and evidence-based priority setting in health.

**Electronic supplementary material:**

The online version of this article (10.1186/s12962-018-0121-z) contains supplementary material, which is available to authorized users.

## Background

Priority setting strategies in the health sector must account for the fact that resources are limited and that tradeoffs are required to decide which health conditions deserve the most attention and which interventions should be used to address them [1, 2]. Even in developed countries, competing investment decisions are influenced by electoral implications, monetary pressures, and divergent ethical perspectives that make significant resource demands on health care systems. The situation is no different in New Zealand (NZ), where the publicly funded health system accounts for over one-fifth of government spending, decentralized across 20 District Health Boards [3, 4]. The NZ Treasury has recently called for allocating health resources more explicitly and sustainably within District Health Boards to deal with the increasing burden of chronic diseases on the aging population and persistent disparities in health outcomes for Māori and Pacific peoples [4].

In an earlier effort to promote efficient and equitable allocation of resources, the National Health Committee advised the NZ Ministry of Health (MoH) from 1993 to 2016 in deciding which technologies and services should be included in the publicly funded health package [5]. This committee approached priority setting in a systematic manner, leveraging criteria of technical and allocative efficiency (as measured by cost-effectiveness) and health equity [6–8]. Instead of depending solely on the judgment of health care professionals, their approach took into consideration the preferences of the general public. However, the prioritization and implementation of the National Health Committee recommendations at the District Health Board level was often slow and cumbersome, with limited customizability for local needs and preferences [9, 10]. In addition, these efforts around health prioritization were focused on health technology assessments, which recognized the importance of evaluating various health technologies/interventions, but did not explicitly prioritize the health conditions themselves to allow for more strategic policymaking [10–12]. In this paper, we present the development and application of an explicit, rational, and comprehensive framework for prioritizing health conditions in alignment with local strategic priorities and public preferences within the Waikato region of NZ.

The Waikato region on the upper North Island of NZ is home to over 400,000 people (almost 10% of the total NZ population). The age profile in this region is similar to the national distribution—approximately 34% of the population is above the age of 50. However, the Waikato region has a larger proportion of Māori than the national average (22.9% vs. 15.8%) and a larger proportion of people in the highest economic deprivation group (25% vs. 20%) [13]. Over 10% of the residents of the Waikato region live in rural areas with low influence from urban employment and without easy access to tertiary care [14]. The Waikato District Health Board (WDHB) is responsible for the health care of all Waikato residents and provides funding for all health services including primary care, pharmaceuticals, community health services, hospital services, research and development, and public health education [15]. In July 2016, the WDHB released its strategy document, “Healthy People Excellent Care” that describes the current public health challenges posed by increasing morbidity due to chronic long-term health conditions, inequities for underserved populations such as Māori who face significantly higher morbidity and mortality rates compared to non-Māori, and increasing demands on the health system from an aging population [16].

To assist WDHB in achieving explicit, transparent, and rational health priority setting in this context, we developed and applied an algorithm to rank the region’s dominant health conditions in order of relative importance, based on a customized set of health criteria. These criteria included measures of disease burden such as disability-adjusted life years (DALYs), economic measures such as cost-effectiveness, and social objectives related to health equity and poverty reduction. Subjective preference weights for each criterion were estimated based on the preferences of the general public in the Waikato region. The goal of this paper is to show how this customizable multi-criteria algorithm can explicitly further a vision of policymaking bodies for rational and transparent priority setting driven by local strategies and preferences.

## Methods

**Fig. 1:**
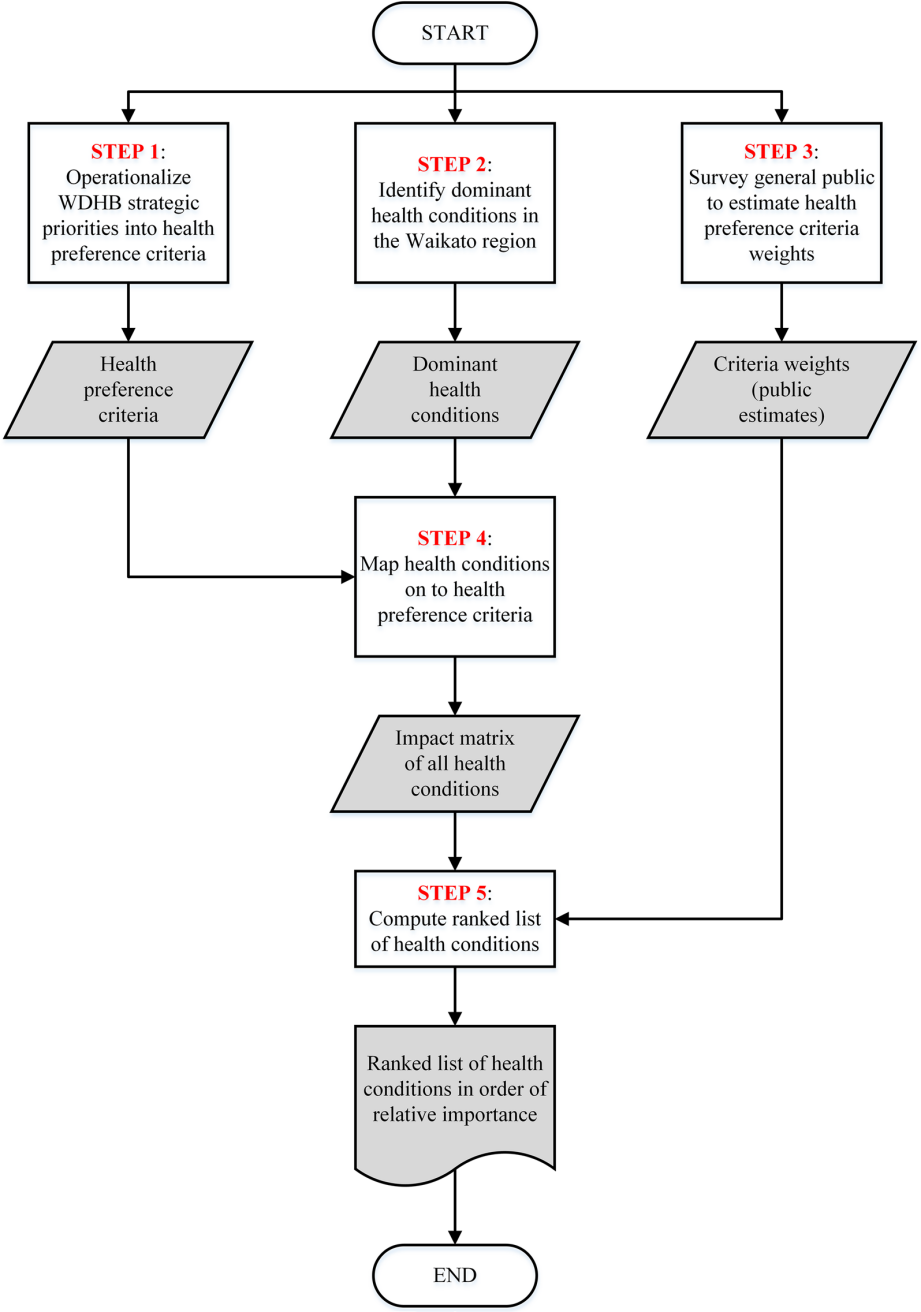
Development and application of a multi-criteria priority setting algorithm in the Waikato region, NZ

As shown in Fig. 1, our overall approach consists of five main steps in which we:

1. Operationalize WDHB strategic priorities into concrete health preference criteria;
2. Identify the dominant health conditions in the Waikato region;
3. Survey the general public in the Waikato region to estimate personal value weights for the health preference criteria;
4. Create an impact matrix of the dominant health conditions mapped to the health preference criteria, based on a systematic review of reports from the NZ MoH, the World Health Organization (WHO), the NZ Treasury, and other published studies from NZ, Australia, the United Kingdom, Canada, and the United States;
5. Compute a priority ranked list of health conditions in order of relative importance using a weighted, additive formula.

### (1) Operationalize WDHB strategic priorities into concrete health preference criteria

**Fig. 2:**
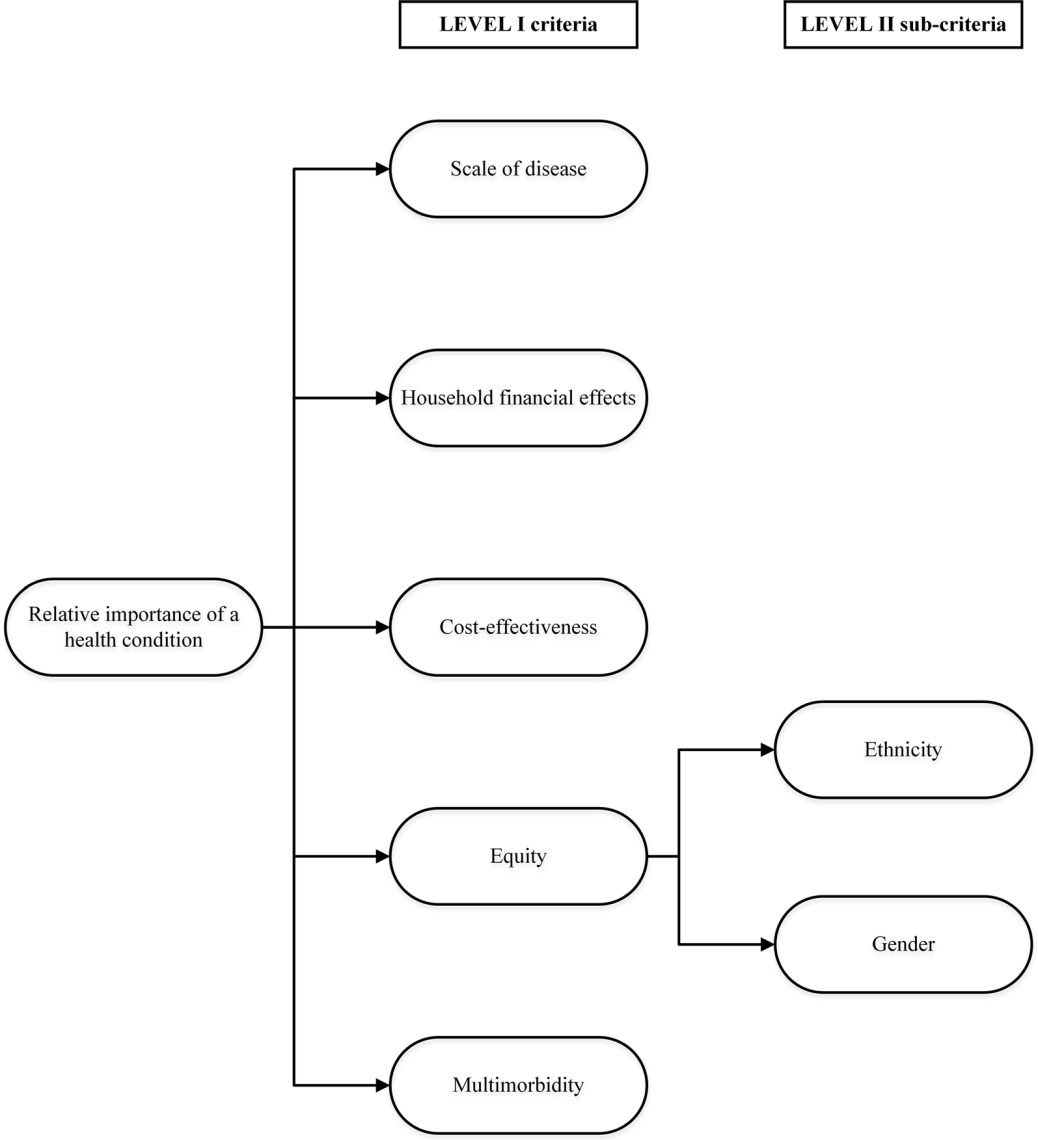
WDHB strategic priorities operationalized into health preference criteria

In collaboration with WDHB executive staff, we identified five health preference criteria (Level I) and two sub-criteria (Level II) that encapsulated the values of WDHB’s strategic vision (Fig. 2) [16]. The health preference criteria are defined as follows:

1. *Scale of disease* What are the morbidity and mortality impacts of the health condition?
2. *Household financial effects* To what extent does the health condition cause personal financial difficulty by reducing earnings or diminishing personal savings?
3. *Cost*-*effectiveness* What is the cost-effectiveness of prevention or treatment methods that are available for the health condition?
4. *Health disparities and inequities* To what extent do vulnerable groups such as women, children, or certain ethnicities carry a disproportionate burden of the health condition? For the purposes of this application, we defined the burden of inequity based on gender and ethnicity sub-criteria (Level II). Rural communities in the Waikato region were not classified as a separate sub-criteria since extensive health data on this group were not available.
5. *Multimorbidity* To what extent does the health condition contribute to a higher burden of multimorbidity?

### (2) Identify the dominant health conditions in the Waikato region

As reported by the NZ MoH in 2014, over three-quarters of the deaths in NZ were attributable to cardiovascular disease, cancers, cerebrovascular disease, respiratory conditions, and mental/behavioral disorders [17]. Diabetes mellitus, maternal/neonatal complications, motor vehicle accidents, and HIV/AIDS were also identified as priority health conditions for the Waikato region based on the NZ MoH 2012 Mortality and Demographic Data Report and the WDHB Health Needs Assessment 2012 [18, 19]. Using this combination of national-level and district-level reporting on the leading causes of mortality and hospitalizations, we selected 25 priority health conditions for input into the priority setting algorithm. The final list was approved by WDHB executive staff to ensure that the dominant health conditions were not only limited to diseases of high mortality, morbidity, or hospitalization rates.

### (3) Survey the general public in the Waikato region to estimate personal value weights for the health preference criteria

Since fair and accountable priority setting practices call for conditions of publicity, relevance, and customizability, we designed and administered a preference elicitation survey such that the relative weights of the health preference criteria defined in Step 1 would be based on personal values expressed by the general public [20].

#### Survey design

We estimated aggregate personal value weights for each of the five health preference criteria based on an anonymous, ordered-choice preference elicitation survey of the general public in the Waikato region. The survey consisted of three parts:

1. Demographic questions: Respondents were asked to select their gender, age range, education, and ethnicity from a list of options validated by WDHB.
2. Ordered choice questions: Value measurements are ideally estimated for all individual criteria and any corresponding sub-criteria, but to minimize the cognitive burden of our survey while maintaining completeness and a more flexible weighting system for the sub-criteria, we limited our survey to the five main criteria (Level I, Fig. 2) [21, 22]. The subsequent allocation of preference weights to the corresponding sub-criteria is detailed in Step 4. Respondents were presented with five hypothetical scenarios representing each of the five health preference criteria (Level I, Fig. 2), and were asked to select the option from an ordered-choice continuum that best reflected how important they personally believed each criterion to be [23]. For example, to estimate household financial effect preferences, respondents were presented with the following question: “A person who is the main income earner in their family is sick and cannot work. The family has to spend some of its weekly budget or savings to get proper treatment for the sick person. How important is it to you to address health conditions that cost families a lot of money due to lost income or increased medical expenses? *Not at all important, A little important, Important, Very important,* or *Extremely important*.”
3. Free-text question: Respondents were asked to provide a free-text answer describing their personal top three health concerns. To assess the validity of our prioritization approach, this information was compared against the relative ranked output list computed by the algorithm.

The survey was written at a Flesch-Kincaid sixth grade reading level (Flesch Reading Ease score = 62.6). The full survey along with a description of the ethnicity and education classification scheme is provided in Additional file 1. The survey was pilot tested during April and May 2017 among 60 patients at Waikato Hospital, a major tertiary care hospital in Hamilton, NZ and was finalized based on resulting recommendations from WDHB staff and patients. The main survey was administered online and on paper to the general public in the Waikato region during June and July 2017.

#### Survey administration

As of June 2017, 6094 (~ 1.5%) Waikato region residents were enrolled in SmartHealth, a virtual health care service newly rolled out by WDHB. During June and July 2017, all SmartHealth enrollees received email invitations to take the anonymous online survey, built on the secure *Qualtrics* platform. To maximize the response rate, two different email subjects were A/B tested among a random half of SmartHealth patients; the subject line that generated a higher click rate was used to invite the remaining half. So that the health preference weights were not solely determined by the subset of patients who seek virtual health care, patients in the main outpatient clinic at the Waikato Hospital were invited to take a paper version of the survey from July 13–21, 2017. All completed paper surveys were entered into the secure *Qualtrics* survey database. Participants of both survey modes were presented with a consent page describing the voluntary and anonymous nature of the survey. Minors (< 18 years of age) were not allowed to participate. The survey met Institutional Review Board exemption criteria per the US Department of Health & Human Services and the Health and Disability Ethics Committee of NZ. Written permission was obtained from WDHB to administer the survey.

#### Health preference criteria weight calculations

In order to calculate the personal value weights for each of the five health preference criteria, the ordered-choice responses from each survey were ranked on a scale of 1–5 (e.g. *Not at all important* received a score of 1 and *Extremely Important* received a score of 5). These numerical values of the survey responses were stored in a preference matrix (A), where P = number of survey respondents and Q = number of health preference criteria (in this case, Q = 5):

$$A \in {\mathbb{R}}^{PXQ} .$$

We explored two different methods of computing aggregate percentage weights for the five health preference criteria:

1. Normalized weights: Preference matrix A was normalized in three steps.
  - Preference values were summed across the entire matrix:
    $$s = \mathop \sum \limits_{i = 1}^{P} \left( {\mathop \sum \limits_{j = 1}^{Q} a_{ij} } \right).$$
  - Each preference value was normalized, dividing it by the sum total:
    $$A^{n} = \left( {{\raise0.7ex\hbox{$1$} \!\mathord{\left/ {\vphantom {1 s}}\right.\kern-0pt} \!\lower0.7ex\hbox{$s$}}} \right) A .$$
  - Normalized preference values for each criterion were summed to create a vector of the percentage trade-off weights:
    $$w\left( {normalized} \right) = \mathop \sum \limits_{i = 1}^{P} a_{i1 }^{n} , \mathop \sum \limits_{i = 1}^{P} a_{i2}^{n} , \ldots \mathop \sum \limits_{i = 1}^{P} a_{iQ}^{n} .$$
2. Rank order centroid (ROC) weights [24]: For this method, we ranked the five criteria in order of importance based on the median preference value of each. The mean preference value was used to break any ties between criteria. A vector of the percentage trade-off weights was then computed using the formula below, setting the number of criteria (n) = 5 and the ranked order of each criterion (j) = 1 through 5.
  $$w_{i} \left( {ROC} \right) = \frac{1}{n}\mathop \sum \limits_{j = 1}^{n} \frac{1}{j} \quad i = 1, \ldots , n .$$

To ensure that the computed percentage weights added up to 100%, we used the Hare–Niemeyer procedure for rounding the weights in both methods [25].

#### Free-text analysis

To calculate the frequencies of the most common health concerns among our survey respondents, we processed free-text responses according to standard text-analysis methods such as excluding common stopwords (e.g. “a”, “the”, “and”, etc.), removing punctuation, and reducing words to their root stem (e.g. “hypertension” or “hypertensive” was reduced to “hypertens”) [26]. From here, we created a term-document matrix, which consisted of one row for every processed free-text word and one column per respondent [27]. Row sums were calculated to yield the overall frequency count for each health concern. If the same word was mentioned more than once by a single respondent, it did not receive multiple counts. To assess the validity of our prioritization approach, health concerns with the highest frequency counts from the term-document matrix were compared against the algorithm output list of health conditions, ranked in order of relative importance.

### (4) Create an impact matrix of the dominant health conditions mapped to the health preference criteria

To quantify the health, economic, and social impacts of each health condition, we created an impact matrix in which scores were assigned to a given health condition for each of the five health preference criteria: scale of disease, household financial effect, cost-effectiveness, health equity, and multimorbidity [28, 29]. Impact scores from 1 to 5 were derived from a systematic review of reports from the NZ MoH, the WHO, the NZ Treasury, and other published studies from NZ, Australia, the United Kingdom, Canada, and the United States. The complete impact matrix of all 25 health conditions mapped to the five health preference criteria is provided in Additional file 2.

#### 1. Scale of disease

We used DALYs for all ages from the WHO Global Health Estimates 2015 summary tables to estimate the morbidity and mortality impact of each of the 25 health conditions identified in Step 2 [30]. The scale of disease impact score for each health condition is defined by its proportional contribution to the total DALYs in NZ (Eq. 1):

- ≥ 5.0% of total NZ DALYs was ranked at level 5.
- ≥ 3.0% and < 5.0% of total NZ DALYs was ranked at level 4.
- ≥ 2.0% and < 3.0% of total NZ DALYs was ranked at level 3.
- ≥ 1.0% and < 2.0% of total NZ DALYs was ranked at level 2.
- < 1.0% of total NZ DALYs was ranked at level 1
  1 $$Scale \;impact\; score = DALY \;Level.$$

#### 2. Household financial effect

Since over 80% of total health expenditure in NZ is from government sources, the personal household financial effect (HFE) was defined in terms of the extent to which household income is diminished by a particular health condition [31]. In December 2015, the NZ Treasury released a working paper that quantified the percentage impact of eight priority health conditions on employment, income support, and personal monthly income [32]. We used these findings to calculate the decrease in personal annual income for a median income earner in NZ with a particular health condition. For health conditions that were not included in the NZ Treasury report or other NZ studies, we used data on decreased income by health condition from Australia, Canada, and the United Kingdom. These high-income Commonwealth countries share comparable health expenditure patterns with NZ, where the total health spending in 2016 was estimated between 9.2 and 10.3% of the total GDP [33]. Quintiles for diminished personal annual income for the median income earner in NZ were calculated using these data gathered for all 25 health conditions. The HFE impact score for each health condition was then calculated based on the quintile ranking of diminished annual income on a scale of 1–5 (Eq. 2):

- > 5091 New Zealand Dollars (NZD) was ranked at level 5.
- > 2789 NZD and ≤ 5091 NZD was ranked at level 4.
- > 1735 NZD and ≤ 2789 NZD was ranked at level 3.
- > 750 NZD and ≤ 1735 NZD was ranked at level 2.
- ≤ 750 NZD was ranked at level 1
  2 $$HFE \;impact \;score = Diminished \;Annual\; Income \;Level.$$

#### 3. Cost-effectiveness

Technically and economically established interventions in NZ were identified for each of the 25 health conditions. The cost-effectiveness (CE) of the primary interventions per health condition was assessed using incremental cost-effectiveness ratios (ICER; NZD per quality-adjusted life year in comparison with no treatment) from the Burden of Disease Epidemiology, Equity and Cost-Effectiveness Programme (BODE^3^), a rich epidemiological database combined with simulated economic models created by the University of Otago in Wellington, NZ [34]. For interventions that were not available in BODE^3^, we used ICER or cost/quality-adjusted life year (QALY) data from the NZ Pharmaceutical Management Agency (PHARMAC), the WHO, the National Institute for Health and Care Excellence (NICE) in the United Kingdom, the Assessing Cost Effectiveness (ACE) Prevention Study in Australia, and other published studies. Historical average conversion rates from 2017 were used to convert ICERs from foreign currencies into NZD [35]. The CE impact score for each intervention pertaining to a corresponding health condition was then calculated by ranking the ICER or cost/QALY data on a scale of 1–5, where more cost-effective interventions received higher scores (Eq. 3). Depending on the availability of data, either the cost-effectiveness of prevention or treatment interventions was used for a given health condition.

- ≤ 5000 NZD was ranked at level 5.
- > 5000 NZD and ≤ 10,000 NZD was ranked at level 4.
- > 10,000 NZD and ≤ 20,000 NZD was ranked at level 3.
- > 20,000 NZD and ≤ 25,000 NZD was ranked at level 2.
- > 25,000 NZD was ranked at level 1
  3 $$CE \;impact\; score = ICER \;or\;\frac{Cost}{QALY}\; Level.$$

#### 4. Health disparities and inequities

We quantified health equity by estimating the burden of each disease across gender and ethnicity, as measured by mortality or prevalence rate ratios. While women in NZ have better health outcomes than men on average, common mental disorders such as anxiety and depression, maternal complications, and breast/reproductive cancers continue to impact women at significant rates [36]. Similarly, while NZ has exhibited rapid health improvements at the macro level (as measured by declines in age-standardized total DALY rates from 1990 to 2015), serious inequities persist by ethnicity and socioeconomic status (SES) [37]. For example, the mortality rate for Māori in 2012 was almost double the non-Māori rate (649.3 vs. 362.0 deaths per 100,000 respectively) [18]. Data published by the NZ MoH also demonstrated that across all SES levels, Māori were more disadvantaged than non-Māori in outcomes related to education, personal income, employment rates, and living conditions [38]. Since ethnicity and SES are so closely linked in NZ, we focused on the burden of disease for Māori compared to non-Māori for the purposes of this study. Burden of disease ratios (i.e. mortality or prevalence rate ratios) by gender and ethnicity were then ranked from 1 to 5:

- > 2.00 was ranked at level 5.
- > 1.51 and ≤ 2.00 was ranked at level 4.
- > 1.26 and ≤ 1.51 was ranked at level 3.
- > 1.11 and ≤ 1.26 was ranked at level 2.
- ≤ 1.11 was ranked at level 1.

Reflecting the poorer health outcomes of Māori across the board, the health equity impact score, defined by the Level II sub-criteria in Fig. 2, was calculated by weighting the burden of disease for ethnicity two times higher than the burden of disease for gender (Eq. 4).

4 $$Equity\; impact \;score \; = \; {\raise0.7ex\hbox{$2$} \!\mathord{\left/ {\vphantom {2 3}}\right.\kern-0pt} \!\lower0.7ex\hbox{$3$}}\; Ethnicity \;Level + {\raise0.7ex\hbox{$1$} \!\mathord{\left/ {\vphantom {1 3}}\right.\kern-0pt} \!\lower0.7ex\hbox{$3$}} \;Gender\; Level.$$

#### 5. Multimorbidity

The coexistence of two or more chronic diseases in a single patient (i.e. multimorbidity) affects a substantial proportion of the general population and is estimated to impact most individuals above the age of 65 [39]. In response to the rising tide of complex chronic diseases and multimorbidity, especially among the elderly, the NZ MoH established *Care Plus*, a funding initiative to improve complex chronic care management [40]. In addition, the Multimorbidity Project at the University of Otago created a multimorbidity (M3) index to predict 1-year mortality, mutually adjusted for 61 chronic conditions [41, 42]. To quantify the average multimorbidity burden associated with each health condition, we summed 1-year mortality log hazard ratios (HR) derived from the M3 index, weighted by multimorbidity prevalence estimates from published literature (see Additional file 2 for details). For a few health conditions that were not present in the M3 index, we used 1-year mortality HRs from other published studies. The multimorbidity impact score was then calculated by ranking the multimorbidity HRs on a scale from 1 to 5 (Eq. 5):

- > 2.00 was ranked at level 5.
- > 1.51 and ≤ 2.00 was ranked at level 4.
- > 1.26 and ≤ 1.51 was ranked at level 3.
- > 1.11 and ≤ 1.26 was ranked at level 2.
- ≤ 1.11 was ranked at level 1
  5 $$Multimorbidity \;impact\; score = Multimorbidity \;Mortality \;HR \;Level .$$

### (5) Compute a priority ranked list of health conditions in order of relative importance using a weighted, additive formula

Using the aggregate health preference values derived from Step 3 to weight the impact matrix from Step 4, a composite algorithm score $(A_{score} )$ was computed for each health condition (Eq. 6). The 25 health conditions were then ranked in order of descending *A*_*score*_ such that greater prioritization values were ascribed to health conditions of higher importance based on multiple criteria, weighted by public opinion.

6 $$A_{score} = {{\left( {w_{s} Scale + w_{h} HFE + w_{c} CE + w_{e} Equity + w_{m} Multimorbidity} \right)} \mathord{\left/ {\vphantom {{\left( {w_{s} Scale + w_{h} HFE + w_{c} CE + w_{e} Equity + w_{m} Multimorbidity} \right)} {100}}} \right. \kern-0pt} {100}}.$$

All analyses were performed using R version 3.2.1 (The R Foundation for Statistical Computing).

## Results

The 25 dominant health conditions selected for input into the algorithm accounted for approximately 70% of the 31,168 total deaths across NZ in 2014 and almost 60% of the 1,059,254 total DALYs in 2015. Of these dominant health conditions, 24 were non-communicable diseases or injuries, with HIV/AIDS being the only communicable disease.

### Demographic profile of survey respondents

A total of 1429 respondents completed the ordered choice survey. Table 1 presents the demographic distribution of respondents by gender, age, education, and ethnicity. The majority of respondents were SmartHealth enrollees (77.3%) and tended to be older females with higher educational levels, compared to the WDHB 2013 census population. The percentage of Māori respondents was identical to the percentage of Māori in the WDHB 2013 census population, but was lower than the estimated proportion of 22.9% in 2017 [13]. While an equal proportion of non-Māori and Māori respondents had college/university degrees (non-Māori = 55.2% and Māori = 56.6%), non-Māori respondents tended to be older (75.4% of non-Māori respondents were 50 years and above compared to 55.3% of Māori respondents, χ^2^ = 38.9, df = 1, p < 0.01).

**Table 1:** Demographic distribution of survey respondents

	% survey respondents	% SmartHealth 2017 population	% WDHB 2013 census population
Gender			
Female	64.3^†^	63.7	52.3
Male	35.1	36.3	47.7
Other/declined	0.6	0.0	0.0
Age^a^			
18–29 years	7.1	20.0	18.0
30–49 years	20.7	29.7	35.3
50–69 years	44.8^††^	34.5	32.7
70+ years	26.9^††^	15.1	14.0
Declined	0.5	0.0	0.0
Education^b^			
None	0.8	–	0.0
Primary school	0.3	–	16.2
Secondary school	32.8	–	43.9
Vocational school	9.6	–	21.7
College/university or higher	55.7^†††^	–	18.2
Declined	0.8	–	0.0
Ethnicity			
European	75.9	69.3	71.5
Māori	16.7	18.4	16.7
Pacific peoples	1.7	1.9	2.8
Asian	2.4	6.2	6.6
Middle Eastern/Latin American/African	1.3	–	0.8
Other/declined	2.0	4.2	1.6

^a^Age percentage categories for the SmartHealth 2017 population add up to slightly less than 100% since minors are allowed to enroll in SmartHealth, but were not allowed to take the survey. Age percentage categories for the WDHB 2013 census population were calculated for individuals 20 years or older

^b^Education-level data were not available for the SmartHealth 2017 population. Education percentage categories for the WDHB 2013 census population were calculated for individuals 20 years or older

^†^The 95% confidence interval estimate of the difference between the female proportion of survey respondents and the female proportion of the WDHB 2013 census population is 9.9–14.9% (χ^2^ = 87.9, df = 1, p < 0.001)

^††^The 95% confidence interval estimate of the difference between the older proportion of survey respondents (≥ 50 years of age) and the older proportion of the WDHB 2013 census population is 23.1–27.7% (χ^2^ = 366.3, df = 1, p < 0.001)

^†††^The 95% confidence interval estimate of the difference between the college educated proportion of survey respondents (college/university education or higher) and the college educated proportion of the WDHB 2013 census population is 35.3–40.5% (χ^2^ = 1341.2, df = 1, p < 0.001)

### Survey response rates

We A/B tested two subject lines for the electronic survey invitation: “A few minutes of your time, please reply by 6 pm tomorrow” and “Help improve healthcare in Waikato”. Respondents who received the subject line that conveyed a sense of urgency responded at a rate one-third higher than respondents who received the more open-ended subject line. The overall response rates by ethnicity for the electronic survey to SmartHealth enrollees were 18.2% for Europeans and 16.3% for Māori. The response rates for the paper surveys could not be calculated since the denominators were unknown (number of patients by ethnicity in the main outpatient clinic at Waikato Hospital could not be estimated).

### Ordered-choice preference profiles

**Fig. 3:**
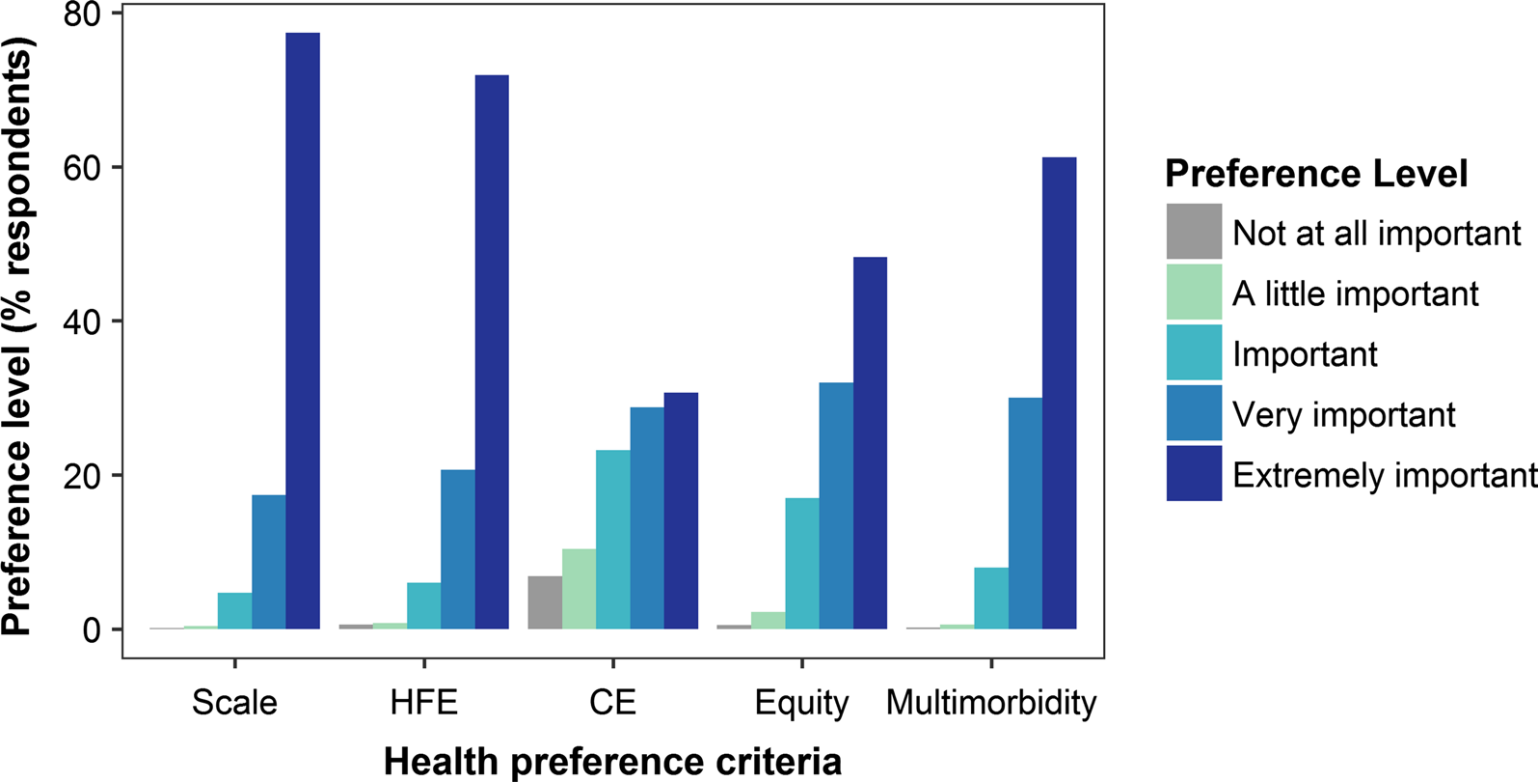
Health preference profiles: ordered-choice preference responses (%) by criteria for all respondents

**Fig. 4:**
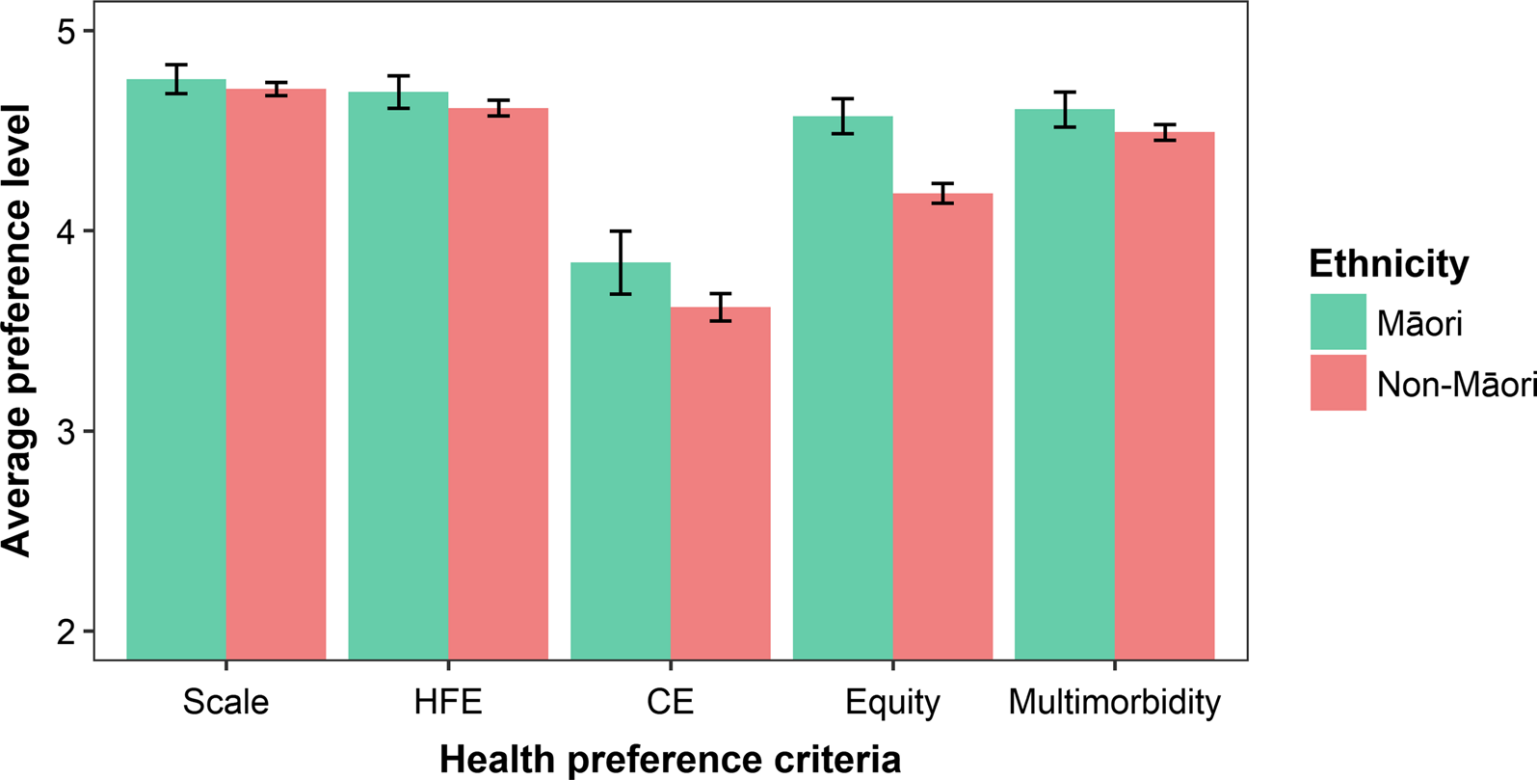
Health preference profiles: average preferences by criteria and ethnicity

Over 70% of respondents ranked Scale and HFE criteria as *Extremely important*, compared to 61.2% for Multimorbidity, 48.3% for Equity, and 30.7% for CE (Fig. 3). Māori respondents had higher average preferences for all criteria compared to non-Māori respondents, especially for Equity and CE (Fig. 4). The average Equity preference was 9.2% higher for Māori (4.6 vs. 4.2, two-sample t-test: t = 7.5324, df = 403.33, p-value < 0.01) and the average CE preference was 6.2% higher (3.8 vs. 3.6, two-sample t-test: t = 2.5534, df = 333.85, p-value = 0.01). Since both these differences for Māori respondents were more than 5%, we computed separate aggregate percentage weights by ethnicity.

**Fig. 5:**
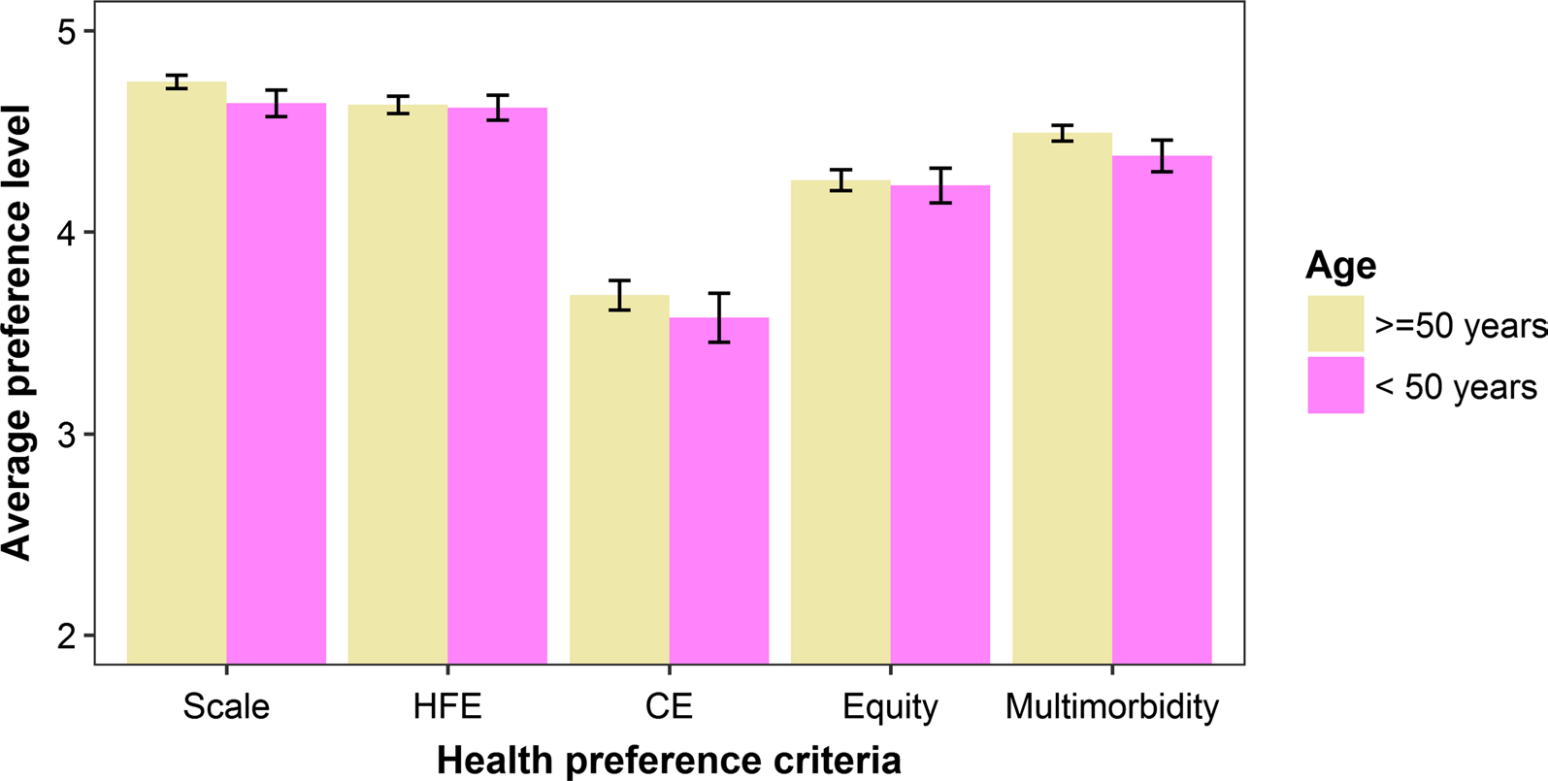
Health preference profiles: Average preferences by criteria and age

Respondents 50 years of age and older also tended to have higher preference profiles compared to younger respondents (Fig. 5). The average Multimorbidity preference was 4.2% higher for older respondents (4.6 vs. 4.4, two-sample t-test: t = − 4.1571, df = 604.17, p-value < 0.01) and the average Scale preference was 2.3% higher (4.7 vs. 4.6, two-sample t-test: t = − 2.8375, df = 607.81, p-value < 0.01). However, since both these differences for older respondents were less than 5%, we did not compute separate percentage weights by age.

### Aggregate health preference criteria weights

The criteria preference weights calculated for the survey respondents using the normalized and ROC weights formulae are shown in Table 2. Since the average preference profile for Māori tended to be higher across all criteria, the weighting for Equity relative to other criteria gained only one extra percentage point for Māori respondents. The order of importance used to calculate the ROC weights for the five criteria was: Scale > HFE > Multimorbidity > Equity > CE. This ordinal ranking did not change by ethnicity, so the ROC weighting presented here is for all respondents. Equity and CE were forced into much smaller ROC percentage weights due to their lower ranks, compared to the normalized weights for these criteria. Given that the average preferences across criteria were near-equal for our survey respondents, the normalized weighting strategy, which also yielded near-equal weights, more consistently represented the preference profile of our survey population compared to weights derived using the ROC methodology. Therefore, to compute the composite algorithm score for each health condition, we used the normalized weights for Māori and non-Māori respondents.

**Table 2:** Health preference criteria weights: normalized vs. ROC

Health preference criteria	Normalized % (non-Māori)	Normalized % (Māori)	ROC % (all respondents)
Scale	22	21	46
HFE	21	21	26
Multimorbidity	21	21	15
Equity	19	20	9
CE	17	17	4

### Ranked list of health conditions using normalized preference weights

The list of 25 priority health conditions in order of relative importance based on their composite algorithm scores is shown in Table 3. Ischaemic heart disease, female breast cancer, and lung cancer were ranked as the top three priority health conditions in the Waikato region. The composite scores were used to establish the ordinal rankings of the health conditions, but it was not meaningful to interpret their relative importance in a multiplicative sense. The ranked lists did not differ appreciably by respondent ethnicity, so the results shown here are for all respondents. Ranked lists by ethnicity are included in Additional file 2.

**Table 3:** Relative importance of health conditions using normalized preference weights (all respondents)

Rank	Health condition	Composite algorithm score
1	Ischaemic heart disease	4.20
2	Female breast cancer	3.84
3	Trachea, bronchus, lung cancer	3.77
4	Suicide	3.71
5	Kidney disease, renal failure	3.58
6	Lymphomas, multiple myeloma	3.41
7	Diabetes	3.35
8	Mouth, oesophagus, and gastric cancer	3.33
9	Premature birth	3.28
10	Dementia	3.25
11	Mental and behavioral disorders	3.23
12	Leukaemia	3.23
13	COPD	3.18
14	Cervical cancer	3.15
15	Cerebrovascular disease	3.11
16	Prostate cancer	3.08
17	Colorectal cancer	3.00
18	Pancreatic cancer	2.85
19	Asthma	2.79
20	Hypertensive disease	2.61
21	HIV/AIDS	2.60
22	Melanoma of skin	2.53
23	Gestational diabetes	2.39
24	Motor vehicle accidents	2.36
25	Peptic ulcer disease	1.89

### Comparison of the normalized ranked list with the free-text health conditions

**Fig. 6:**
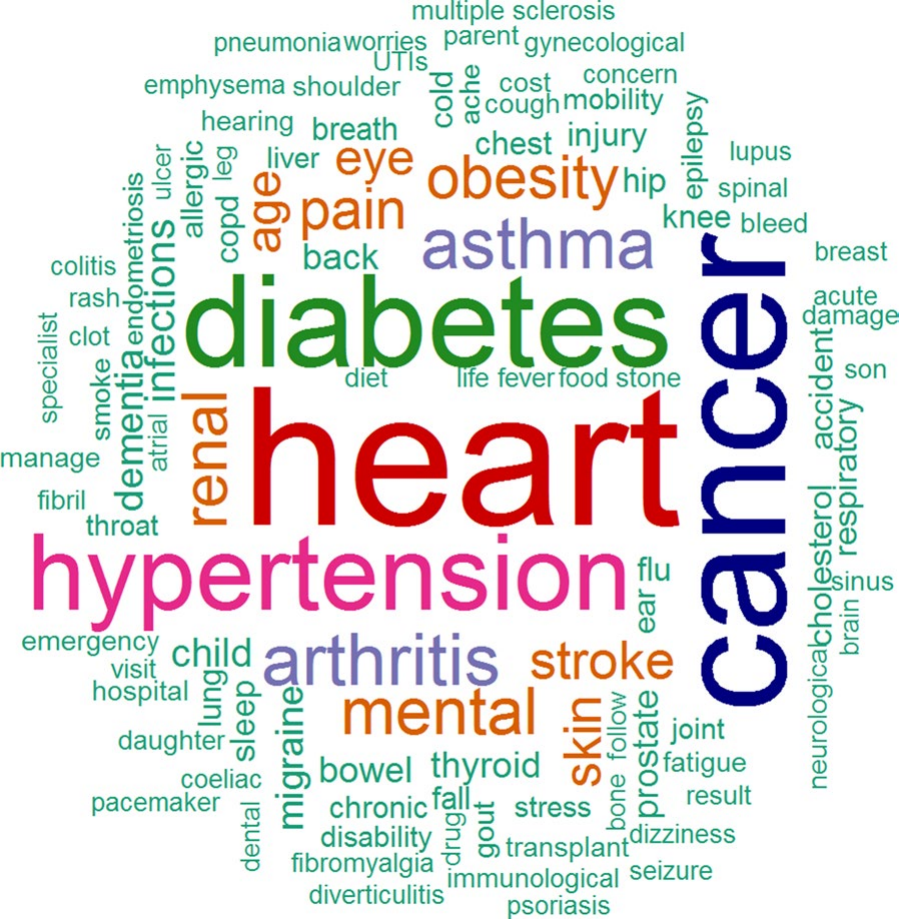
Free-text frequency word cloud for top 100 health conditions of concern

**Fig. 7:**
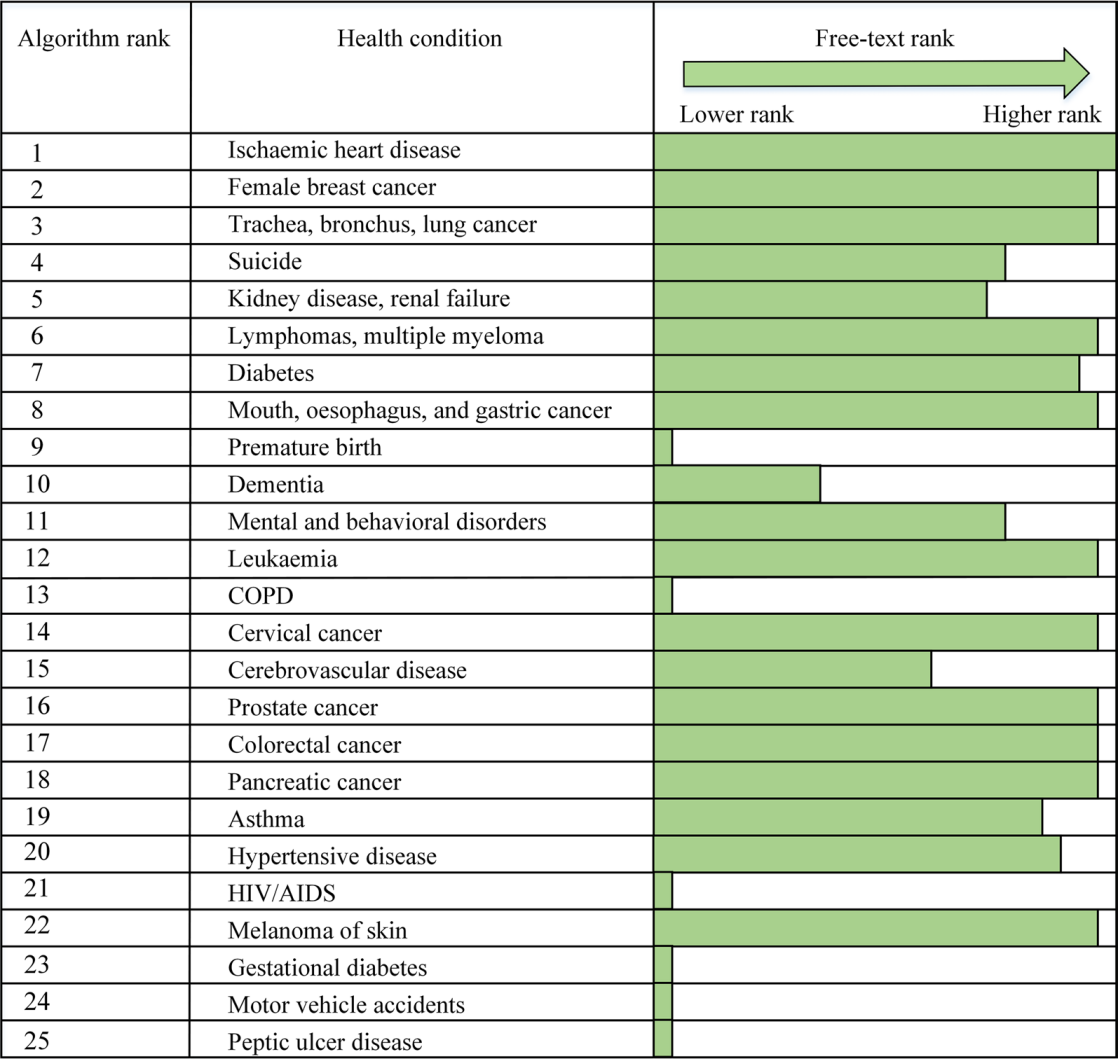
Comparison of algorithm ranking with free-text ranking. The free-text ranking presented here is for the top 100 health conditions of concern, based on the term-document matrix created from survey responses. For visual clarity, the exact numeric ranking of the free-text health concerns is represented by a proportionally scaled arrow

The top 100 health conditions of concern listed by the survey respondents are shown in Fig. 6. Except for “arthritis”, “obesity”, and “pain”, the first ten diseases of concern listed by the survey respondents were included in the list of WDHB priority health conditions. In close alignment with our algorithm, heart disease, cancers, and diabetes, were the top three broad health concerns of the survey respondents. Respondents did not appreciably differentiate between types of cancers, so we could not reliably compare the algorithm rankings and free-text rankings by type of cancer. On the other hand, mental and cognitive disorders such as suicide and dementia were ranked within the top ten health conditions by our algorithm, above asthma and hypertension which were explicitly identified as higher priority health conditions according to the free-text responses (Fig. 7). Premature birth and chronic obstructive pulmonary disease (COPD) also received higher rankings from our algorithm, but were not listed with appreciable frequency in the free-text responses.

## Discussion

The development and application of our multi-criteria algorithm closely follows the best practices recommended by the International Society For Pharmacoeconomics and Outcomes Research (ISPOR) Task Force on multiple criteria decision analysis [28]. This strategic priority setting algorithm allowed us to estimate what health conditions should be prioritized and why [12]. To address these priority health conditions, a follow-up tactical approach, such as health technology assessment, can be used to inform which interventions should be appropriately funded [43]. In order to generate our ranked list of strategically prioritized health conditions, we elicited the underlying health preference weights and pressing health concerns of the general public while building on detailed, preexisting datasets pertaining to the morbidity and mortality rates, financial impacts, and burdens of inequity of various health conditions in NZ. According to the Corruption Perceptions Index 2016, NZ ranks second out of 176 countries in the world [44]. This exceptionally high level of data transparency made it possible for us to demonstrate a direct, modeled approach to determining which health conditions are most important to address, while quantifying a link between five, near-equal underlying priorities that drive the health concerns of the general public in the Waikato region.

In this discussion, we provide three recommendations for applying our algorithm to strategic health priority setting. First, to achieve accountable, relevant, and transparent health care priority setting, the public perspective can be used to weigh public health evidence across multiple criteria [45]. We measured the preferences of the general public in the Waikato region to estimate health preference criteria weights and to validate the algorithm output against the free-text responses. Our findings suggest that there is broad correspondence and alignment between the algorithm output and the explicit health concerns of the public. Certain health conditions are of concern to the public not just because of the morbidity and mortality they cause, but also because of household financial effects, multimorbidity risks, and burdens of inequity. However, a majority of the free-text responses were in broader, more general bins than our algorithm output list, so fine-grained comparability with the algorithm ranking was not possible. Future iterations of this approach might incorporate more clinical specificity in asking respondents to specify the types of cancers or chronic diseases that concern them the most. Misalignments between the public free-text health concerns and the algorithm output list can help policymakers identify specific health conditions that are rare, underdiagnosed, or lack public awareness. For example, our algorithm ranked suicide as the fourth most important health outcome of concern, but since young Māori males—who were underrepresented in our survey—are at a particularly high risk of suicide, our findings suggest that awareness of suicide and its risk factors in NZ might be lacking [46]. Similarly, COPD was ranked higher than asthma and hypertensive disorders even though the latter two conditions were listed as higher priority according to our survey respondents. This misalignment supports the fact that while asthma in NZ receives considerable attention and publicity, COPD is often underdiagnosed and less well understood [47]. In consultation with WDHB, our analysis primarily focused on higher burden diseases in the Waikato region. To ensure that orphan health conditions also receive appropriate attention, future iterations of this study might be expanded to include rarer, high impact diseases.

Our second finding is that estimating health preferences of the general public is only as strong as the preference elicitation and subsequent weighting methodologies that are used. We recommend that the ROC weighting method be used if only the ordinal ranking between criteria is available or if the raw multi-criteria preferences are substantially different from each other. If raw multi-criteria preferences are near-equal (as expressed by our survey population), normalized weights more consistently capture this information compared to ROC weights, which are forced into a much wider range across criteria. In our survey population, Māori respondents ranked concerns of health equity higher than non-Māori respondents. However, since Māori respondents also tended to rank the other health preference criteria higher than non-Māori respondents did, the resulting normalized weights for the five health preference criteria were not appreciably different between both groups, with health equity carrying a near-equal percentage weighting along with scale of disease, household financial effects, and multimorbidity. As a result, lung cancer, which has a mortality rate three times higher among Māori compared to non-Māori and has arguably not received enough public health emphasis due to factors of stigma and blame, was ranked third in the algorithm output list, close to breast cancer and bowel cancer—diseases that have traditionally received more attention in NZ [48, 49]. It is unclear from our study whether the comparable percentage weightings of all five health preference criteria underscored the relatively equal importance they represented for our survey respondents or whether the survey questions were sensitive enough to measure the respondents’ true preferences. How a survey question is framed can skew public perception, especially if there is no opportunity for broader clarification and discourse with survey respondents regarding accurate and precise definitions of the criteria under question. For example, while it might not be surprising that cost-effectiveness received the lowest ranking of the five health preference criteria, public opinion would likely be more favorable to incorporating considerations of cost-effectiveness into priority setting if the burden of cost-ineffective treatments was presented as the direct responsibility of private taxpayers vs. the indirect responsibility of public hospitals.

Therefore, our third recommendation is that our algorithm output along with the survey of underlying preferences and explicit health concerns of the general public are meant to inform, not replace, representative and deliberative priority setting processes. Our survey response rate was approximately 18%, in line with the expectation that internet-based surveys without incentives or multimodal follow-up typically yield response rates less than 25% [50, 51]. The survey population mirrored the ethnic distribution in the broader Waikato region, but respondents were more likely to be older females with higher education levels compared to the general Waikato population. Our survey and algorithm results highlight the importance of soliciting diverse preferences using multiple modalities that are not limited by literacy, especially when underserved populations are among the primary stakeholders. Fair and representative policymaking will ideally be based on concepts of deliberative democracy, in which representative groups of stakeholders interpret the algorithm output in the light of multiple competing special interests, through transparent, in-person, and iterative discourse. These deliberative processes will ideally consider how the preferences of relevant groups in the general public vary from each other, what levels of diversity might be involved in the deliberative discourse, and how these decisions will influence policy outcomes [52].

This study can be improved by expanding the range of non-redundant health preference criteria to more accurately capture the broader impact of ill-health. For example, foregone income alone due to disease burden is likely an underestimate of household financial effects. We considered lost income due to absenteeism, but did not explicitly include estimates of presenteeism or loss of productivity from working while ill [53]. Similarly, over 7% of Waikato region residents reported looking after a household member who was ill or had a disability, but estimates for caretaking costs were not included in this study [54]. While we considered the 1-year mortality impact of average multimorbidity estimates, we did not account for the overlap between the scale of disease criterion and multimorbidity, nor did we consider the downstream social impacts of certain health conditions (e.g. stigma associated with mental health conditions, HIV/AIDS, etc.). Finally, survey respondents in our study were not fully representative of the broader Waikato population. Young men with lower education levels were especially underrepresented in our study. Pacific peoples, like the Māori, face significant health inequities but were not weighted separately in our dataset because of the small number of Pacific respondents. Future applications of this priority setting approach might include a survey with stratified sampling and multimodal follow-up strategies to improve response rates and to minimize sampling bias. Study design can also include contextualized community-based participatory research methodologies such as deliberative polling, in which the full range of criteria scales and algorithm inputs/outputs might be iterated among representative stakeholders [55–57].

As mentioned earlier, the main strengths of this study come from leveraging public health preferences to weight a detailed dataset relevant to the NZ context. The preference elicitation methodology we used is relatively easy to implement and analyze. Operationalizing strategic priorities and estimating criteria weights from the general public in this manner can be replicated by other public health institutions with the goal of understanding which health conditions deserve the most attention and why.

## Conclusions

We developed and applied a strategic priority setting approach in the Waikato region of NZ, in which preexisting disease indicators were combined in an algorithm with public preferences to quantify the relative importance of dominant health conditions. Policymakers can use this two-pronged approach not only to determine the immediate health needs of their constituency, but even to predict the importance of new health conditions as they emerge. Ideally in the context of democratic deliberation, future applications of this model can be extended to developed and developing countries alike, where scarce health care resources must be more efficiently and equitably allocated.

## Additional files

**Additional file 1.** WDHB preference elicitation survey and ethnicity/education classification schemes for survey respondents.

**Additional file 2.** Impact Matrix scores for each health condition and relative importance of health conditions by ethnicity.

## Abbreviations

ACE: assessing cost effectiveness prevention study
BODE^3^: Burden of Disease Epidemiology, Equity and Cost-Effectiveness Programme
CE: cost-effectiveness
COPD: chronic obstructive pulmonary disease
DALY: disability-adjusted life years
HFE: household financial effects
HR: hazard ratio
ICER: incremental cost-effectiveness ratio
ISPOR: International Society For Pharmacoeconomics and Outcomes Research
M3: Multimorbidity Index
MoH: Ministry of Health
NICE: National Institute for Health and Care Excellence
NZ: New Zealand
NZD: New Zealand Dollars
PHARMAC: New Zealand Pharmaceutical Management Agency
QALY: quality-adjusted life years
ROC: rank order centroid
SES: socioeconomic status
WDHB: Waikato District Health Board
WHO: World Health Organization
