# Supplementary material for: Priority setting in health: development and application of a multi-criteria algorithm for the population of New Zealand’s Waikato region
Source: Cost Eff Resour Alloc. 2018 Nov 9;16(Suppl 1):52. doi: 10.1186/s12962-018-0121-z (PMC6225550; doi:10.1186/s12962-018-0121-z)
Supplement: Supplementary file 1 — Additional file 1. WDHB preference elicitation survey and ethnicity/education classification schemes for survey respondents. [file 12962_2018_121_MOESM1_ESM.pdf]

Waikato District Health Board (WDHB), the Māori health team at WDHB, and the Harvard T.H. Chan School of Public Health would like you to complete this short survey.

1. What is this survey being used for?
WDHB serves almost 400,000 people and we will use results from this survey to improve the nature, quality, and accessibility of our health care services.

2. How long will this survey take?
This survey should not take more than 10 minutes.

3. Do I need to provide any personal information?
We are not asking for any information that could be used to identify you.

4. How old do I have to be to complete this survey?
You must be 18 years of age or older to participate.

5. Are there any costs to complete this survey?
There are no costs for you to participate.

6. Am I required to participate?
Your participation is totally voluntary and you can stop at any time.

7. Who can I contact with questions about this survey?
For more information about this survey, please contact Rashmi Dayalu at rdayalu@hsph.harvard.edu. You may keep this top page for your records.

Kia ora! Thank you very much for your time.

Instructions: In the questions that follow, please select your answers using ✓ or ✗ in the circle provided. For example:

1. What is your gender?
- ○ Male
- ✓ Female
- ○ Other

OR

1. What is your gender?
- ○ Male
- ✗ Female
- ○ Other

1. What is your gender?

- ⬭ Male
- ⬭ Female
- ⬭ Other

2. What is your age?

- ⬭ 18-29 years
- ⬭ 30-49 years
- ⬭ 50-69 years
- ⬭ 70+ years

3. What is your highest level of education?

- ⬭ None
- ⬭ Primary school
- ⬭ Secondary school
- ⬭ Vocational school
- ⬭ College / University or higher

4. Which ethnic group(s) do you belong to? Choose all answers that apply.

- ⬭ New Zealand European
- ⬭ Māori
- ⬭ Samoan
- ⬭ Cook Island Māori
- ⬭ Tongan
- ⬭ Niuean
- ⬭ Chinese
- ⬭ Indian
- ⬭ Other (such as Dutch, Japanese, Tokelauan) (specify): ______________________________

Please continue to PAGE 2.

The next two pages give you a few brief examples followed by questions that will help us understand which health problems you think are the most important to address in the Waikato District.

5. A person has no appetite for food, feels nauseous or sick, and is so weak that they need to spend most of the day in bed. In this context, they suffer from a disability because they cannot function normally. Over a long period of time, these health problems may put the individual at a higher risk of death.
How important is it to you to address health conditions that cause disability or death?

- ◯ Extremely important
- ◯ Very important
- ◯ Important
- ◯ A little important
- ◯ Not at all important

6. A person who is the main income earner in their family is sick and cannot work. The family has to spend some of its weekly budget or savings to get proper treatment for the sick person.
How important is it to you to address health conditions that cost families a lot of money due to lost income or increased medical expenses?

- ◯ Extremely important
- ◯ Very important
- ◯ Important
- ◯ A little important
- ◯ Not at all important

7. A public hospital must decide between offering a highly effective, expensive drug vs. a somewhat effective, lower-cost drug used to treat a particular health condition.
How important is it to you for public hospitals to think about cost in addition to effectiveness of health care treatments?

- ◯ Extremely important
- ◯ Very important
- ◯ Important
- ◯ A little important
- ◯ Not at all important

Please continue to PAGE 3.

8. People with poor nutrition and crowded housing are more likely to get some diseases at an earlier age (for example, rheumatic fever, heart disease, or diabetes) compared to people who have better nutrition and live in less crowded homes.
How important is it to you to address health conditions that are more likely to affect underprivileged groups of people?

- Extremely important
- Very important
- Important
- A little important
- Not at all important

9. Conditions such as untreated high blood pressure or diabetes can develop into heart disease or kidney disease. Combined health problems like this can decrease a person's quality of life or increase their risk of death.
How important is it to you to address health conditions that might develop into additional health problems for an individual?

- Extremely important
- Very important
- Important
- A little important
- Not at all important

10. List three health problems that worry you or other members of your family the most:

i.

ii.

iii.

Ethnicity and Education classification schemes

Ethnicity: Procedures for data collection and output of ethnicity data in New Zealand are standardized per the New Zealand Ministry of Health.[1] Ethnicity data presented in this paper were classified into six ethnic groups according to a modified version of the Ministry's Level 1 classification scheme: European, Māori, Pacific peoples, Asian, Middle Eastern/Latin American/African, and Other/Declined. Respondents who self-identified as Māori were always classified as Māori, regardless of any other additional ethnic categories they might have selected. Respondents who identified with multiple, non-Māori ethnic categories were classified according to their minority ethnic category of choice (e.g. if a respondent selected New Zealand European and Samoan, they were classified as Pacific peoples). Finally, respondents who listed an "Other" category that couldn't be reclassified into any of the other five groups or declined to provide an ethnicity response were included in the Other/Declined category.

Education: Our preference elicitation survey asked respondents to select their highest level of education from a list of five options: None, Primary school, Secondary school, Vocational school, and College/University or higher. The New Zealand Qualifications Framework (NZQF) defines standardized benchmarks for all secondary and tertiary educational qualifications in New Zealand.[2] To compare the education levels of the survey respondents with the expected distribution from WDHB 2013 census data, the education levels in the survey were mapped against NZQF as shown in Table 1A.[3]

Table 1A. Preference elicitation survey education categories by WDHB 2013 census categories (NZQF)

**Survey education categories**	**WDHB 2013 census categories (NZQF)**
None	No qualification
Primary school	No qualification
Secondary school	Levels 1, 2, and 3
Vocational school	Levels 4 Certificate and Levels 5/6 Diploma
College / University or higher	Levels 7 and above

[1] Ethnicity Data Protocols for Health and Disability Sector: https://www.health.govt.nz/publication/hiso-100012017-ethnicity-data-protocols

[2] New Zealand Qualifications Framework: https://education.govt.nz/ministry-of-education/our-role-and-our-people/education-in-nz/#Primary

[3] http://archive.stats.govt.nz/Census/2013-census/data-tables/dhb-tables.aspx
