# Supplementary material for: Priority setting in health: development and application of a multi-criteria algorithm for the population of New Zealand’s Waikato region
Source: Cost Eff Resour Alloc. 2018 Nov 9;16(Suppl 1):52. doi: 10.1186/s12962-018-0121-z (PMC6225550; doi:10.1186/s12962-018-0121-z)
Supplement: Supplementary file 2 — Additional file 2. Impact Matrix scores for each health condition and relative importance of health conditions by ethnicity. [file 12962_2018_121_MOESM2_ESM.pdf]

APPENDIX 2: Impact Matrix

The Impact Matrix contains impact scores of 25 dominant health conditions in New Zealand (NZ) mapped to five health preference criteria: scale of disease, household financial effect, cost-effectiveness, health inequity, and multimorbidity. Impact scores were derived from a systematic review of reports from the NZ Ministry of Health, the World Health Organization (WHO), the NZ Treasury, and other published studies from NZ, Australia, the United Kingdom, Canada, and the United States.

Table 2A. Impact Matrix of 25 dominant health conditions in NZ: Impact scores were ranked from 1 to 5 (in order of lowest to highest impact/priority)

	**Health preference criteria**					
Health Condition	*Scale of disease*[1]	*Household financial effect (HFE)*[2]	*Cost-effectiveness (CE)*	*Health inequity*[3]		*Multimorbidity*[4]
				Ethnicity	*Gender*	
Ischaemic heart disease	5	3	5	4	1	5
Female breast cancer	3	4	4	4	5	4
Trachea, bronchus, lung cancer	4	3	3	5	1	5
Suicide	3	5	3	3	1	5
Kidney, renal disease	2	5	2	5	1	5
Lymphomas, multiple myeloma	2	4	3	4	1	5
Diabetes	4	2	3	5	1	4
Mouth, oesophagus, and gastric cancer	2	3	3	5	1	5
Premature birth	2	5	1	4	1	5
Dementia	3	5	1	1	3	5
Mental and behavioral disorders	5	1	4	5	3	2

[1] DALY estimates from the WHO Global Health Estimates 2015 summary tables were used to estimate the morbidity and mortality impacts of the corresponding health conditions in NZ, unless otherwise mentioned [67].
[2] Proportion of negative impact on average monthly earnings was derived from Dixon 2015, unless otherwise mentioned [58].
[3] Maori/non-Maori and female/male mortality rate ratios were referenced from the latest NZ Ministry of Health mortality reports, unless otherwise mentioned [68,69].
[4] Average multimorbidity burdens associated with each health condition were estimated using the M3 index from Stanley 2017, unless otherwise mentioned [70].

Leukaemia	1	4	4	3	1	5
COPD	4	1	2	5	1	5
Cervical cancer	1	2	3	5	5	5
Cerebrovascular disease	4	5	1	2	2	3
Prostate cancer	2	2	5	2	1	5
Colorectal cancer	3	3	4	1	1	4
Pancreatic cancer	1	3	3	3	1	5
Asthma	3	2	3	3	3	3
Hypertensive disease	1	1	5	5	1	3
HIV/AIDS	1	4	3	1	1	4
Melanoma of skin	2	1	5	1	1	4
Gestational diabetes	1	2	1	5	5	3
Motor vehicle accidents	2	4	1	5	1	1
Peptic ulcer disease	1	1	2	5	1	2

1. Ischaemic heart disease:

- The CE score is based on PHARMAC, 2001 estimates for statins in NZ [1].
- The M3 multimorbidity score was calculated using average multimorbidity prevalence estimates associated with heart failure [2].

2. Female breast cancer:

- The CE score is based on BODE^3 estimates for female breast cancer [3].
- The M3 multimorbidity score was calculated using average multimorbidity prevalence estimates associated with female breast cancer [4].

3. Trachea, bronchus, lung cancer:

- The HFE score is based on household financial costs associated with lung, bronchus, or trachea cancer, estimated by the University of Bristol, 2013 [5].
- The CE score is based on BODE^3 estimates for lung, trachea, and bronchus cancers [3].
- The M3 multimorbidity score was calculated using average multimorbidity prevalence estimates associated with lung cancer [6].

4. Suicide:

- Age-specific suicide rates in New Zealand in 2013 show that the median age at suicide was approximately 40 years. Assuming a median weekly income of 621 NZD, the foregone annual income per individual to suicide was estimated at 32,401 NZD.
- The NZ Ministry of Health launched a national campaign at address the issue of suicide from multiple angles of prevention [7]. The CE score here is based on the higher end of very cost-effective interventions for individuals in Australia who make non-fatal suicide attempts [8].
- The health inequity score of suicide by ethnicity and gender was based on estimates from the NZ Ministry of Health [9].
- The multimorbidity score for suicide attempters was based on estimates of comorbid mental conditions such as severe anxiety, depression, and substance disorders [10].

5. Kidney, renal disease:

- The HFE score is based on productivity/income losses associated with changes in employment for predialysis and dialysis patients in NZ and end-stage renal patients in Canada [11,12].
- The CE score is based on a systematic review of economic evaluations of haemodialysis for people with end-stage renal failure [13].
- The health inequity score of kidney disease and renal failure by ethnicity and gender was based on estimates from the NZ and Australia [14,15].
- The M3 multimorbidity score was calculated using average multimorbidity prevalence estimates associated with chronic kidney disease in the United States [16].

6. Lymphomas, multiple myeloma:

- The HFE score is based on household financial costs associated with lymphoma, leukaemia, or myeloma, estimated by the University of Bristol, 2013 [5].
- The CE score is based on $BODE^3$ estimates for Hodgkin’s lymphoma, non-Hodgkin’s lymphoma, and myeloma [3].
- The M3 multimorbidity score was calculated using average multimorbidity prevalence estimates associated with non-Hodgkin lymphoma in the United States [17].

7. Diabetes:

- The CE score is based on PHARMAC, 2005 estimates for insulin analogues in NZ [18].
- The M3 multimorbidity score was calculated using average multimorbidity prevalence estimates associated with diabetes in the United States [19].

8. Mouth, oesophagus, and gastric cancer:

- The HFE score is based on household financial costs associated with oesophagus, stomach, pancreas, or liver cancers estimated by the University of Bristol, 2013 [5].
- The CE score is based on average BODE^3 estimates for lip, mouth, pharynx, oesophageal, and stomach cancers [3].
- The M3 multimorbidity score was calculated using average multimorbidity prevalence estimates associated with gastric cancer in the United States [20].

9. Premature birth:

- The HFE score is estimated from socioeconomic attainments for individuals in the Helsinki Birth Cohort Study who were born late-preterm (34-36 weeks) and were 34% more likely to earn in the lowest tercile range [21]. To compute the corresponding HFE of preterm birth in NZ, we assumed uniform distribution within each quintile bin of NZ earnings [22]. Weekly personal income ranges for each tercile were calculated using piecewise linear models of the relevant quintiles. For example, to estimate the income range of the lowest tercile (33%), we calculated the slope and y-intercept of income for individuals in the second quintile (21% - 40%). These parameters were then used to estimate the upper bound of the income range for individuals in the 33rd percentile, from which the average HFE score for premature birth was calculated.
- The CE score for regular midwifery visits/staffing is based on NICE estimates from the United Kingdom [23].
- The multimorbidity score for premature birth was based on prevalence estimates for various medical disabilities at birth in Norway [24].

10. Dementia:

- The scale of impact score was from the Alzheimers New Zealand 2016 report [25].
- The HFE score was computed from productivity losses, reduced employment, and absenteeism estimates in NZ associated with dementia [25].
- The CE score is based on dementia screening tests in primary care in the United Kingdom [26].

- The health inequity score of dementia by ethnicity and gender was based on estimates from the Alzheimers New Zealand 2016 report [25].
- The M3 multimorbidity score was calculated using average multimorbidity prevalence estimates associated with dementia in Spain [27].

11. Mental and behavioral disorders:
    - The HFE score was computed from productivity losses, reduced employment, and absenteeism estimates associated with mental/behavioral disorders [28,29]. To estimate the impact of various mental health conditions on work/school and social/family life, the World Health Organization World Mental Health Survey Initiative version of the Composite International Diagnostic Interview (CIDI 3.0) was conducted among 12,992 respondents in New Zealand. Respondents reported mild-moderate effects of anxiety disorders on work/school functioning and moderate-marked effects of mood disorders on work/school functioning. To quantify the indirect costs of mental health conditions in New Zealand, we used the OECD 2014 mental health report that estimated a 16.7 % higher absenteeism rate for individuals with moderate disorders compared to individuals with no mental health disorder from among 21 European OECD countries in 2010. Assuming this higher rate for individuals with mental health conditions (4.8 days vs. 4.1 days), and assuming that 45% of all lost working days were due to mental illness (2.2 days), this translates to a 1% negative impact on average annual earnings.
    - The CE score for mental/behavioral disorders is based on screenings/interventions for individuals in Australia [30].
    - The health inequity score by ethnicity and gender for common mental/behavioral disorders was based on NZ Ministry of Health statistics. In the 2014, the New Zealand Mental Health foundation released a report that women were 1.6 times more likely receive a diagnosis of a common mental health condition compared to men (20% vs. 13%) [31]. According to the Office of the Director of Mental Health Annual Report (2015), Maori individuals account for 26% of all mental health service users even though they make up only approximately 16% of the New Zealand population [32]. On average, Maori were 3.5 times more likely than non-Maori to be subject to compulsory treatment orders. While the over-representation of Maori in compulsory treatment is a complex issue pertaining to cultural biases, disproportionately harsh treatment of Maori with mental health conditions, and lack of Maori family/community involvement in mental health treatment, we are using these rates as a proxy to demonstrate the higher burden of mental health conditions among Maori.
    - The M3 multimorbidity score was calculated using the average M3 of anxiety/behavioral disorders and major psychiatric disorders from Stanley 2017 combined with physical comorbidity prevalence estimates associated with anxiety disorders [33].

12. Leukaemia:
    - The HFE score is based on household financial costs associated with lymphoma, leukaemia, or myeloma, estimated by the University of Bristol, 2013 [5].
    - The CE score is based on BODE³ estimates for leukaemia [3].
    - The M3 multimorbidity score was calculated using average multimorbidity prevalence estimates associated with acute myeloid leukaemia in Germany [34].

13. COPD:
    - The CE score for COPD is based on long-term air humidification therapy estimates in NZ [35].
    - The M3 multimorbidity score was calculated using average multimorbidity prevalence estimates associated with COPD in the United States and Spain [36].

14. Cervical cancer:
    - The HFE score is based on household financial costs associated with cervical, ovarian, or uterine cancers estimated by the University of Bristol, 2013 [5].
    - The CE score is based on BODE³ estimates for cervical cancer [3].
    - The M3 multimorbidity score was calculated using average multimorbidity prevalence estimates associated with cervical cancer in Italy [37].

15. Cerebrovascular disease:
    - The CE score for cerebrovascular disease is based on cost-effectiveness of acute stroke units in NZ [38].
    - The M3 multimorbidity score was calculated using average multimorbidity prevalence estimates associated with cerebrovascular disease in Turkey [39].

16. Prostate cancer:
    - The CE score is based on BODE³ estimates for prostate cancer [3].
    - The M3 multimorbidity score was calculated using average multimorbidity prevalence estimates associated with prostate cancer in the United States [40].

17. Colorectal cancer:
    - The HFE score is based on household financial costs associated with colorectal cancer estimated by the University of Bristol, 2013 [5].
    - The CE score is based on BODE³ estimates for colorectal cancer [3].
    - The M3 multimorbidity score was calculated using average multimorbidity prevalence estimates associated with colorectal cancer in the Netherlands [41].

18. Pancreatic cancer:
    - The HFE score is based on household financial costs associated with oesophagus, stomach, pancreas, or liver cancers estimated by the University of Bristol, 2013 [5].
    - The CE score is based on BODE³ estimates for pancreatic cancer [3].
    - The M3 multimorbidity score was calculated using average multimorbidity prevalence estimates associated with pancreatic cancer in Denmark [42].

19. Asthma:
    - The HFE score is based on direct household financial costs associated with asthma in NZ [43].
    - The CE score for chronic asthma is based on cost-effectiveness of salmeterol xinafoate/fluticasone propionate combination inhalers in the United Kingdom [44].
    - The health inequity score of asthma by ethnicity and gender was based on estimates from the NZ Ministry of Health [45].
    - The M3 multimorbidity score was calculated using average multimorbidity prevalence estimates associated with asthma [46].

20. Hypertensive disease:
    - Obesity in NZ is a primary risk factor for hypertension [47]. While the economic effect of hypertension on worker productivity/income has not been quantified in New Zealand, lost productivity for overweight and obesity in both New Zealand and Australia has been documented [48]. According to the Australian Statistics Bureau in 2004-05, employees who were overweight/obese had absenteeism rates that were 14.3% higher than employees who were underweight or normal weight [49]. We used the Moodie et al. findings and the New Zealand Treasury 2010 report to estimate that the average absenteeism rate for overweight/obese employees was 0.5 days more than the average absenteeism rate for underweight/normal weight employees in New Zealand (4.6 days vs. 4.1 days) [48,50]. Assuming an average 8-hour work day and 2,087 average work hours per year, this translates to a 0.2% negative impact on average annual income [51].

- The CE score is based on PHARMAC estimates for pulmonary arterial hypertension interventions in NZ [1].
- The M3 multimorbidity score was calculated using average multimorbidity prevalence estimates associated with hypertension in Denmark [52].

21. HIV/AIDS:
    - The HFE score is based on direct household financial impacts associated with HIV/AIDS in NZ [53].
    - The CE score is based on PHARMAC estimates for HAART in NZ [1].
    - The health inequity score of HIV/AIDS by ethnicity and gender was based on estimates from the NZ AIDS Foundation [54].
    - The M3 multimorbidity score was calculated using average multimorbidity prevalence estimates associated with HIV/AIDS in the United Kingdom [55].

22. Melanoma of skin:
    - The CE score is based on BODE^3 estimates for melanoma [3].
    - The M3 multimorbidity score was calculated using average multimorbidity prevalence estimates associated with melanoma in the United States [56].

23. Gestational diabetes:
    - DALYs for all maternal conditions in NZ was used as a proxy for DALYs for gestational diabetes.
    - According to the National Institutes of Health in the United States, women with a history of gestational diabetes (GDM) have up to a 60% chance of developing diabetes in the next 1-2 decades of life [57]. Therefore, we used the impact of diabetes on average monthly earnings (5.4% negative impact) as a proxy for the impact of gestational diabetes on average monthly earnings [58].
    - The CE score for regular midwifery visits/staffing is based on NICE estimates from the United Kingdom [23].
    - The health inequity score of gestational diabetes by ethnicity was based on estimates from the NZ Ministry of Health [59].
    - The M3 multimorbidity score was calculated using average multimorbidity prevalence estimates associated with diabetes in the United States [19].

24. Motor vehicle accidents:
- The CE score is based on road injury prevention measures in Europe [60].
- The M3 multimorbidity score was calculated using average multimorbidity prevalence estimates associated with traumatic brain injury in the United States and Finland [61,62].

25. Peptic ulcer disease:
- The HFE score is based on work loss due to peptic ulcer disease in the United States [63].
- The CE score for peptic ulcer disease based on a model of *H. pylori* screening in NZ [64].
- The health inequity score of peptic ulcer disease was based on proxy of the incidence of upper gastrointestinal haemorrhage by ethnicity in NZ and by gender in Denmark [65,66].

Table 3A. Relative importance of health conditions using normalized preference weights (Non- Māori respondents vs. Māori respondents)

	Non-Māori respondents		Māori respondents	
	**Health condition**	**Score**	**Health condition**	**Score**
1	Ischaemic heart disease	4.20	Ischaemic heart disease	4.18
2	Female breast cancer	3.84	Female breast cancer	3.86
3	Trachea, bronchus, lung cancer	3.77	Trachea, bronchus, lung cancer	3.76
4	Suicide	3.71	Suicide	3.71
5	Kidney disease, renal failure	3.58	Kidney disease, renal failure	3.59
6	Lymphomas, multiple myeloma	3.41	Lymphomas, multiple myeloma	3.42
7	Diabetes	3.35	Diabetes	3.34
8	Mouth, oesophagus, and gastric cancer	3.33	Mouth, oesophagus, and gastric cancer	3.34
9	Premature birth	3.28	Premature birth	3.29
10	Dementia	3.25	Leukaemia	3.25
11	Mental and behavioral disorders	3.23	Dementia	3.23
12	Leukaemia	3.23	Mental and behavioral disorders	3.23
13	COPD	3.18	Cervical cancer	3.19
14	Cervical cancer	3.15	COPD	3.17
15	Cerebrovascular disease	3.11	Cerebrovascular disease	3.09
16	Prostate cancer	3.08	Prostate cancer	3.07
17	Colorectal cancer	3.00	Colorectal cancer	2.98
18	Pancreatic cancer	2.85	Pancreatic cancer	2.87
19	Asthma	2.79	Asthma	2.79
20	Hypertensive disease	2.61	Hypertensive disease	2.63
21	HIV/AIDS	2.60	HIV/AIDS	2.60
22	Melanoma of skin	2.53	Melanoma of skin	2.52
23	Gestational diabetes	2.39	Gestational diabetes	2.43
24	Motor vehicle accidents	2.36	Motor vehicle accidents	2.37
25	Peptic ulcer disease	1.89	Peptic ulcer disease	1.91

References:

1. Metcalfe S. Updated cost utility analysis for statins. PHARMAC [Internet]. 2001;(January):1–11. Available from: https://www.pharmac.govt.nz/assets/statins-cua-updated-2001-01-30.pdf
2. Murad K, Goff DC, Morgan TM, Burke GL, Bartz TM, Kizer JR, et al. Burden of Comorbidities and Functional and Cognitive Impairments in Elderly Patients at the Initial Diagnosis of Heart Failure and Their Impact on Total Mortality. The Cardiovascular Health Study. JACC Hear Fail. 2015;3(7):542–50.
3. Burden of Disease Epidemiology, Equity & Cost-Effectiveness Programme (BODE^3) [Internet]. University of Otago; [cited 2017 Oct 8]. Available from: http://www.otago.ac.nz/wellington/departments/publichealth/research/bode3/index.html
4. Fu M, Axelrod D, Guth A, Cleland C, Ryan C, Weaver K, et al. Comorbidities and Quality of Life among Breast Cancer Survivors: A Prospective Study. J Pers Med [Internet]. 2015;5(3):229–42. Available from: http://www.mdpi.com/2075-4426/5/3/229/
5. The financial impacts of cancer [Internet]. 2013. Available from: http://www.bristol.ac.uk/geography/research/pfrc/themes/fincap/the-financial-impacts-of-cancer/
6. Islam KMM, Jiang X, Anggondowati T, Lin G, Ganti AK. Comorbidity and Survival in Lung Cancer Patients. Cancer Epidemiol Biomarkers Prev [Internet]. 2015;24(7):1079–85. Available from: http://cebp.aacrjournals.org/cgi/doi/10.1158/1055-9965.EPI-15-0036
7. The New Zealand Suicide Prevention Strategy 2006 - 2016 [Internet]. 2006. Available from: http://www.moh.govt.nz/suicideprevention
8. Cost-effectiveness of suicide prevention interventions [Internet]. Assessing Cost-Effectiveness in Prevention (ACE). 2010. Available from: https://public-health.uq.edu.au/files/655/ACE-P_pamphlet_16.pdf
9. Suicide Facts: Deaths and intentional self harm hospitalisations, 2013 [Internet]. Wellington; 2016. Available from: https://www.health.govt.nz/publication/suicide-facts-deaths-and-intentional-self-harm-hospitalisations-2013
10. Nock M, Hwang I, Sampson N, Kessler R. Mental disorders, comorbidity and suicidal behavior: results from the National Comorbidity Survey Replication. Mol Psychiatry [Internet]. 2009;15(8):868–76. Available from: http://www.nature.com/mp/journal/v15/n8/abs/mp200929a.html
11. Walker RC, Howard K, Tong A, Palmer SC, Marshall MR, Morton RL. The economic considerations of patients and caregivers in choice of dialysis modality. Hemodial Int. 2016;20(4):634–42.
12. Zelmer JL. The economic burden of end-stage renal disease in Canada. Kidney Int [Internet]. 2007;72(0085–2538 (Print)):1122–9. Available from: http://dx.doi.org/10.1038/sj.ki.5002459
13. Mowatt G, Vale L, Perez J, Wyness L, Fraser C, MacLeod A, et al. Systematic review of the effectiveness and cost-effectiveness, and economic evaluation, of home versus hospital or satellite unit haemodialysis for people with end-stage renal failure. Heath Technol Assess. 2003;7(2):1–174.
14. Collins JF. Kidney disease in Maori and Pacific people in New Zealand. Clin Nephrol [Internet]. 2010;74 Suppl 1:S61-5.

Available from: http://www.ncbi.nlm.nih.gov/pubmed/20979966
15. Stewart J. End-stage renal failure appears earlier in men than in women with polycystic kidney disease. Am J Kidney Dis. 1994;24(2):181–3.
16. Stevens LA, Li S, Wang C, Huang C, Becker BN, Bomback AS, et al. Prevalence of CKD and Comorbid Illness in Elderly Patients in the United States: Results From the Kidney Early Evaluation Program (KEEP). Am J Kidney Dis. 2010;55(3 SUPPL. 2).
17. Hester L, Park SI, Lund JL. Patterns of comorbidity among older U.S. patients with non-Hodgkin lymphoma. J Clin Oncol. 2016;34(7_suppl):304–304.
18. PHARMAC. PHARMAC responds on long-acting insulin analogues. N Z Med J [Internet]. 2005;118(1224):1–9. Available from: https://www.pharmac.govt.nz/assets/nzmj-2005-10-28-pharmac-responds-on-long-acting-insulin-analogues.pdf
19. Iglay K, Hannachi H, Joseph Howie P, Xu J, Li X, Engel SS, et al. Prevalence and co-prevalence of comorbidities among patients with type 2 diabetes mellitus. Curr Med Res Opin [Internet]. 2016;32(7):1243–52. Available from: http://www.tandfonline.com/doi/full/10.1185/03007995.2016.1168291
20. Danese MD, Gleeson ML, Langeberg WJ, Ke J, Kelsh MA. Prevalence and incidence of comorbidities associated with gastric cancer. J Clin Oncol [Internet]. 2014;32(3_suppl):39–39. Available from: http://ascopubs.org.ezp-prod1.hul.harvard.edu/doi/abs/10.1200/jco.2014.32.3_suppl.39
21. Heinonen K, Eriksson JG, Kajantie E, Pesonen A-K, Barker DJ, Osmond C, et al. Late-Preterm Birth and Lifetime Socioeconomic Attainments: The Helsinki Birth Cohort Study. Pediatrics [Internet]. 2013;132(4):647–55. Available from: http://pediatrics.aappublications.org/cgi/doi/10.1542/peds.2013-0951
22. 2013 quickstats about income [Internet]. Statistics New Zealand. 2013 [cited 2018 Mar 5]. Available from: http://archive.stats.govt.nz/Census/2013-census/profile-and-summary-reports/quickstats-income.aspx
23. Cookson G, Jones S, Vlymen J Van. The Cost-Effectiveness of Midwifery Staffing and Skill Mix on Maternity Outcomes [Internet]. 2014. Available from: https://www.nice.org.uk/guidance/ng4/evidence/economic-evaluation-report-pdf-5277277
24. Moster D, Lie RT, Markestad T. Long-Term Medical and Social Consequences of Preterm Birth. N Engl J Med [Internet]. 2008;359(3):262–73. Available from: http://www.nejm.org/doi/abs/10.1056/NEJMoa0706475
25. Deloitte. Dementia Economic Impact Report 2016. Alzheimers New Zeal [Internet]. 2017;(March):1–78. Available from: http://www.alzheimers.org.nz/getmedia/79f7fd09-93fe-43b0-a837-771027bb23c0/Economic-Impacts-of-Dementia-2017.pdf/
26. Tong T, Thokala P, McMillan B, Ghosh R, Brazier J. Cost effectiveness of using cognitive screening tests for detecting dementia and mild cognitive impairment in primary care. Int J Geriatr Psychiatry. 2017;32(12):1392–400.
27. Poblador-Plou B, Calderón-Larrañaga A, Marta-Moreno J, Hancco-Saavedra J, Sicras-Mainar A, Soljak M, et al. Comorbidity of dementia: a cross-sectional study of primary care older patients. BMC Psychiatry [Internet]. 2014;14(1):84. Available from: http://bmcpsychiatry.biomedcentral.com/articles/10.1186/1471-244X-14-84
28. Deloitte UK. At a tipping point ? Workplace mental health and wellbeing. 2017;(March).

29. OECD. Sick on the Job? Myths and Realities about Mental Health and Work, Mental Health and Work [Internet]. OECD Publishing; 2012. 214 p. Available from: http://dx.doi.org/10.1787/9789264124523-en
30. Vos T, Carter R, Barendregt J, Mihalopoulos C, Veerman L, Magnus A, et al. Assessing Cost-Effectiveness in Prevention [Internet]. 2010. 1-122 p. Available from: https://public-health.uq.edu.au/research/centres/past-centres/assessing-cost-effectiveness-ace-prevention-study
31. The Ministry of Health New Zealand. Mental Health Foundation : Quick Facts and Stats 2014. 2014;1–10. Available from: https://www.mentalhealth.org.nz/assets/Uploads/MHF-Quick-facts-and-stats-FINAL.pdf
32. Office of the Director of Mental Health Annual Report 2013 [Internet]. 2014. Available from: http://www.health.qld.gov.au/mha2000/documents/annual-report-2013.pdf
33. Belik SL, Sareen J, Stein MB. Anxiety Disorders and Physical Comorbidity. Oxford Handb Anxiety Relat Disord. 2008;(October 2017):1–29.
34. Wass M, Hitz F, Schaffrath J, Müller-Tidow C, Müller LP. Value of different comorbidity indices for predicting outcome in patients with acute myeloid leukemia. PLoS One. 2016;11(10):1–13.
35. Milne RJ, Hockey H, Rea H. Long-term air humidification therapy is cost-effective for patients with moderate or severe chronic obstructive pulmonary disease or bronchiectasis. Value Heal [Internet]. 2014;17(4):320–7. Available from: http://dx.doi.org/10.1016/j.jval.2014.01.007
36. Divo M, Cote C, De Torres JP, Casanova C, Marin JM, Pinto-Plata V, et al. Comorbidities and risk of mortality in patients with chronic obstructive pulmonary disease. Am J Respir Crit Care Med. 2012;186(2):155–61.
37. Ferrandina G, Lucidi A, Paglia A, Corrado G, MacChia G, Tagliaferri L, et al. Role of comorbidities in locally advanced cervical cancer patients administered preoperative chemoradiation: Impact on outcome and treatment-related complications. Eur J Surg Oncol [Internet]. 2012;38(3):238–44. Available from: http://dx.doi.org/10.1016/j.ejso.2011.12.001
38. Ao B, P. B, Feigin V, Anderson C. Are Stroke Units Cost Effective? Evidence from a New Zealand Stroke Incidence and Population-Based Study. Int J Stroke. 2012;7(8):623–30.
39. Karatepe AG, Gunaydin R, Kaya T, Turkmen G. Comorbidity in patients after stroke: Impact on functional outcome. J Rehabil Med. 2008;40(10):831–5.
40. Xiao H, Tan F, Goovaerts P, Ali A, Adunlin G, Huang Y, et al. Construction of a Comorbidity Index for Prostate Cancer Patients Linking State Cancer Registry with Inpatient and Outpatient Data. J Registry Manag. 2013;40(4):159–64.
41. Van Leersum NJ, Janssen-Heijnen MLG, Wouters MWJM, Rutten HJT, Coebergh JW, Tollenaar RAEM, et al. Increasing prevalence of comorbidity in patients with colorectal cancer in the South of the Netherlands 1995-2010. Int J Cancer. 2013;132(9):2157–63.
42. Bang UC, Benfield T, Hyldstrup L, Bendtsen F, Beck Jensen JE. Mortality, cancer, and comorbidities associated with chronic pancreatitis: A Danish nationwide matched-cohort study. Gastroenterology. 2014;146(4):989–94.
43. Holt S, Beasley R. The burden of asthma in New Zealand. Asthma and Respiratory Foundation of New Zealand. 2001.

44. Doull I, Price D, Thomas M, Hawkins N, Stamuli E, Tabberer M, et al. Cost-effectiveness of salmeterol xinafoate/fluticasone propionate combination inhaler in chronic asthma. Curr Med Res Opin [Internet]. 2007;23(5):1147–59. Available from: http://www.tandfonline.com/doi/full/10.1185/030079907X187982
45. Respiratory disease | Ministry of Health NZ [Internet]. [cited 2018 Mar 6]. Available from: https://www.health.govt.nz/our-work/populations/maori-health/tatau-kahukura-maori-health-statistics/nga-mana-hauora-tutohu-health-status-indicators/respiratory-disease
46. Su X, Ren Y, Li M, Zhao X, Kong L, Kang J. Prevalence of comorbidities in asthma and nonasthma patients. Med (United States). 2016;95(22):1–7.
47. Mclean RM, Williams S, Mann JI, Miller JC, Parnell WR. Blood pressure and hypertension in New Zealand: results from the 2008/09 Adult Nutrition Survery. N Z Med J. 2013;126(1372):66–79.
48. Lal A, Moodie M, Ashton T, Siahpush M, Swinburn B. Health care and lost productivity costs of overweight and obesity in New Zealand. Aust N Z J Public Health. 2012;36(6):550–6.
49. Overweight and Obesity in Adults, Australia, 2004-05 [Internet]. Australian Bureau of Statistics. c=AU; o=Commonwealth of Australia; ou=Australian Bureau of Statistics; 2008 [cited 2018 Mar 6]. Available from: http://www.abs.gov.au/AUSSTATS/abs@.nsf/Latestproducts/4719.0Main Features62004-05?opendocument&tabname=Summary&prodno=4719.0&issue=2004-05&num=&view=
50. Holt H. The Cost of Ill Health. 2010.
51. Computing Hourly Rates of Pay Using the 2,087-Hour Divisor [Internet]. [cited 2018 Mar 6]. Available from: https://www.opm.gov/policy-data-oversight/pay-leave/pay-administration/fact-sheets/computing-hourly-rates-of-pay-using-the-2087-hour-divisor/
52. Paulsen MS, Andersen M, Thomsen JL, Schroll H, Larsen P V., Lykkegaard J, et al. Multimorbidity and blood pressure control in 37 651 hypertensive patients from Danish general practice. J Am Heart Assoc. 2013;2(1):19–23.
53. Grierson J, Pitts M, Whyte M, Misson S, Hughes A, Saxton P, et al. Living with HIV in New Zealand: balancing health and quality of life. N Z Med J. 2004;117(1200):1–9.
54. HIV in New Zealand | New Zealand AIDS Foundation [Internet]. [cited 2018 Mar 6]. Available from: https://www.nzaf.org.nz/hiv-aids-stis/hiv-aids/hiv-in-new-zealand/
55. Lorenc A, Ananthavarathan P, Lorigan J, Banarsee R, Jowata M, Brook G. The prevalence of comorbidities among people living with HIV in Brent: A diverse London Borough. London. J Prim Care. 2014;6(4):84–90.
56. Lee EH, Nijhawan RI, Nehal KS, Dusza SW, Levine A, Hill A, et al. Comorbidity assessment in skin cancer patients: A pilot study comparing medical interview with a patient-reported questionnaire. J Skin Cancer. 2015;2015.
57. Preventing Type 2 Diabetes | NIDDK [Internet]. [cited 2018 Mar 6]. Available from: https://www.niddk.nih.gov/health-information/diabetes/overview/preventing-type-2-diabetes
58. Dixon S. The Employment and Income Effects of Eight Chronic and Acute Health Conditions [Internet]. 2015. Available from:

http://purl.oclc.org/nzt/p-1811
59. White C, Reid S, Damiris V. Māori health literacy research: Gestational diabetes mellitus [Internet]. 2014. Available from: http://www.moh.govt.nz/notebook/nbbooks.nsf/0/F6CDB34BBE65334CCC257D10006C7300/$file/GDM-report-FINAL.pdf
60. Polinder S, Toet H, Panneman M, van Beeck E. Methodological Approaches for Cost–effectiveness and Cost–utility Analysis of Injury Prevention Measures [Internet]. 2011. Available from: http://www.euro.who.int/__data/assets/pdf_file/0007/144196/e95096.pdf
61. Holcomb EM, Millis SR, Hanks RA. Comorbid disease in persons with traumatic brain injury: Descriptive findings using the modified cumulative illness rating scale. Arch Phys Med Rehabil [Internet]. 2012;93(8):1338–42. Available from: http://dx.doi.org/10.1016/j.apmr.2012.04.029
62. Raj R, Kaprio J, Korja M, Mikkonen ED, Jousilahti P, Siironen J. Risk of hospitalization with neurodegenerative disease after moderate-to-severe traumatic brain injury in the working-age population: A retrospective cohort study using the Finnish national health registries. PLoS Med. 2017;14(7):1–16.
63. Henke CJ, Levin TR, Henning JM, Potter LP. Work loss costs due to peptic ulcer disease and gastroesophageal reflux disease in a Health Maintenance Organization. Am J Gastroenterol [Internet]. 2000;95(3):788–92. Available from: http://www.nature.com/doifinder/10.1111/j.1572-0241.2000.01861.x
64. Teng AM, Kvizhinadze G, Nair N, McLeod M, Wilson N, Blakely T. A screening program to test and treat for Helicobacter pylori infection: Cost-utility analysis by age, sex and ethnicity. BMC Infect Dis. 2017;17(1):1–11.
65. Irwin J, Ferguson R, Weilert F, Smith A. Incidence of upper gastrointestinal haemorrhage in Maori and New Zealand European ethnic groups, 2001-2010. Intern Med J. 2014;44(8):735–41.
66. Rosenstock SJ, Jørgensen T. Prevalence and incidence of peptic ulcer disease in a Danish County--a prospective cohort study. Gut. 1995;36(6):819–24.
67. WHO | Global Health Estimates [Internet]. WHO. World Health Organization; 2018 [cited 2018 Feb 22]. Available from: http://www.who.int/healthinfo/global_burden_disease/estimates/en/index2.html
68. New Zealand Ministry of Health. Mortality and Demographic Data 2012 [Internet]. 2012. 95 p. Available from: http://www.health.govt.nz/publication/mortality-and-demographic-data-2012
69. New Zealand Ministry of Health. Cancer : New registrations and deaths 2013. 2016. 19-93 p.
70. Stanley J, Sarfati D, Chb MB. The new Measuring Multimorbidity index predicted mortality better than Charlson and Elixhauser indices amongst the general population. J Clin Epidemiol [Internet]. 2017; Available from: http://dx.doi.org/10.1016/j.jclinepi.2017.08.005
